# LncRNA AK089514/miR-125b-5p/TRAF6 axis mediates macrophage polarization in allergic asthma

**DOI:** 10.1186/s12890-023-02339-1

**Published:** 2023-01-30

**Authors:** Xiaolong Zhu, Ling He, Xueqin Li, Weiya Pei, Hui Yang, Min Zhong, Mengying Zhang, Kun Lv, Yingying Zhang

**Affiliations:** 1 Central Laboratory, The First Affiliated Hospital of Wannan Medical College (Yijishan Hospital of Wannan Medical College), 2 Zheshan Western Road, Wuhu, 241001 People’s Republic of China; 2 Key Laboratory of Non-Coding RNA Transformation Research of Anhui Higher Education Institutes (Wannan Medical College), Wuhu, 241001 People’s Republic of China; 3 Non-Coding RNA Research Center of Wannan Medical College, Wuhu, 241001 China; 4 Department of Blood Transfusion of Yijishan Hospital, The First Affiliated Hospital of Wannan Medical College, Wuhu, 241001 Anhui China; 5 Department of Laboratory Medicine (Wannan Medical College), Wuhu, 241001 China; 6 Anhui Province Clinical Research Center for Critical Respiratory Medicine, Wuhu, 241001 China

**Keywords:** miRNA, Allergic asthma, Macrophage polarization, ceRNA

## Abstract

**Background:**

Micro RNA (miRNA) plays important roles in macrophage polarization. However, the manner in which miRNA regulate macrophage polarization in response to dermatophagoides farinae protein 1(Der f1)-induced asthma has not been defined. This study aims to explore the role of miRNAs in regulating macrophages in asthma.

**Methods:**

The microRNAs which may regulate asthma were selectd by Microarrays. The function of miR-125b-5p in macrophage and Der f1-induced asthma were detected in vivo experiment. The long non coding RNA (lncRNA) AK089514/miR-125b-5p/TRAF6 axis was predicted by bioinformatics and confirmed by dual luciferase reporter assay.

**Results:**

In this study, we found that miR-125b-5p is highly expressed in M2 macrophages and bronchoalveolar lavage fluid (BALF) cells with Der f1-induced asthma. In response to the challenge of Der f1, miR-125b-5p KD attenuated allergic airway inflammation of mice by preventing M2 macrophages polarization. Mechanistic studies indicated that lncRNA AK089514 functioned as a competing endogenous RNA for miR-125b-5p, thereby leading to the depression of its endogenous target TNF receptor associated factor 6 (TRAF6).

**Conclusions:**

miR-125b-5p is significantly over-expressed in asthma, and AK089514–miR-125b-5p–TRAF6 axis play critical role in asthma by modulating macrophage polarization. Our findings may provide a potential new target for potential therapeutic and diagnostic target in asthma.

**Supplementary Information:**

The online version contains supplementary material available at 10.1186/s12890-023-02339-1.

## Introduction

Asthma is an inflammatory pulmonary disease that characterized by infiltration of a variety of inflammatory cells into the airway submucosa. The downstream events, eosinophilic airway infiltration, peribronchial goblet cell hyperplasia, and chronic airway remodeling which mediated by immune cells are considered the major characteristics of asthma. It affects people of all ages and has become a serious public health threat worldwide [1–3]. Asthma is a heterogeneous disease characterized by distinct phenotypes. The most common of them are T-helper type 2 (Th2) inflammation that characterized by eosinophilic inflammation. Th2-type may be allergic or non allergic. There is also a non T2 asthma that characterized by neutrophils inflammation [4]. As one of the most abundant immune cell populations in the lung, alternatively activated (M2) macrophages play important roles in allergic asthma mainly through type 2 (Th2) responses [5, 6].

Macrophages are very versatile, as local microenvironment shape their phenotypic and functional properties [7]. Based upon stimuli, macrophages were activated to distinct subtypes which is a revisable process called polarization. The classic “M1” proinflammatory macrophages are characterized by elaboration copious amounts of inflammatory cytokines (tumor necrosis factor TNF-α, interleukin IL-12, IL6, and NO) and increased microbicidal activity. The distinct subtypes of “M2” anti-inflammatory macrophages, secrete high levels of anti-inflammatory cytokines (IL-4, IL-5, IL-10, IL-13) and are characterized by increased arginase (Arg) activity, participating in tissue remodeling, resolution of inflammation, atopic disease, parasite response [8, 9], and scavenger functions [10, 11]. Pathology is usually related to dynamic changes of macrophage polarization, with M1 macrophages associated with initiating and sustaining acute inflammation and M2 macrophages involved in resolution or smoldering chronic inflammation [12–15]. Thus, macrophage polarization is an important immunological event in the progression of various disease, such as infection, autoimmune disease, atherosclerosis, cancer, and insulin resistance [16–18]. Therefore, uncovering the mechanisms of macrophage plasticity and polarization will benefit for macrophage-centered diagnostic and therapeutic strategies in asthma.

The crucial role of epigenetic mechanism in the pathogenesis of allergic asthma has been demonstrate well [19]. Non-coding RNAs (ncRNAs) regulation, the main epigenetic mechanisms, is a field that is under intensive investigation. An accumulating body of research shows that long non-coding RNAs (lncRNAs) and microRNAs (miRNAs) play important roles in gene regulation of immune system [20]. It is reported that miR-511-3p and miR-155 play an important role in allergic asthma by regulating macrophage activation [21]. In addition to miRNAs, recent studies have found that lncRNAs are involved in the differentiation process of mammalian innate and adaptive immune cells [22]. Moreover, multiple lncRNAs including BAZ2B, GAS5, and Mirt2 has been revealed to be involved in immunologic diseases and cancer through macrophage activation [23–25]. In our previous work, we using microarrays observed that miRNA-155 and -125b-5p were upregulated in M2 macrophages [26]. In this study, we aimed to investigate the effects and mechanisms of miR-125b-5p on macrophage polarization in asthma. MiRNA-125b-5p was highly expressed in M2 macrophages and promoted the polarization of macrophages to M2. In allergic asthma model mice induced by Der f1, miR-125b-5p deficient attenuated allergic airway inflammation, revealed that miR-125b-5p plays a critical role in asthma. Mechanistic studies demostrated that lncRNA AK089514 could competitively bind miR-125b-5p to affect TRAF6 expression. Importantly, our research provides a new theoretical and experimental basis for the pathogenesis and biological prevention of AA.

## Materials and methods

### Animals

BALB/c and C57BL/6 mice (Six-week-old, 16–20 g) were purchased from the Experimental Animal Center of Qinglongshan (Nanjing, China) and raised in a specific-pathogen-free (SPF) mouse colony. All animal experiments were completely in accord with all RRIVE guidelines (https://arriveguidelines.org) for the reporting of animal experiments. The Animal Ethics Committee of the Yijishan Hospital of Wannan Medical College approved the protocols of all animal experiment. The Ethical code for animal study is AECYJS-2019011.

### BMDMs isolation and culture

Bone marrow-derived macrophages (BMDMs) were isolated from the femurs and tibias of mice. The isolated cells were suspended in Dulbecco’s modified Eagle’s medium (DMEM, Gibco, Life Technologies, NY, USA) supplemented with 20% fetal bovine serum (FBS, Gibco) and 20% supernatant of L929 cells. Then BMDMs were added in 6-well plate at a density of 1 × 10^6^ cells per well and incubated at 37 °C with 5% CO_2_ (M0). 7 days later, the BMDMs cells were incubated in fresh RPMI-1640 medium (10% FBS). After 24 h of culture, the BMDMs cells were polarized by treating with DMEM medium (10% FBS) supplemented with 100 ng/ml LPS plus 20 ng/ml IFN-γ (for M1 polarization) or 20 ng/ml IL-4 (for M2 polarization).

### Transfection of miRNAs, siRNAs and lncRNA smart silencer

BMDMs were transfected with 100 nm miR-125b-5p mimic, miR-125b-5p inhibitor or lncRNA AK089514 smart silencer using riboFECT™ CP (RiBoBio, Guangzhou, China) according to the manufacturer’s instructions. miR-125b-5p mimic, miR-125b-5p inhibitor and lncRNA AK089514 smart silencer were synthesized by RiboBio Co. (Guangzhou, China). The scrambled sequence was used as a negative control (NC). 72 h after transfection, the cells were used for the following experiments.

### Real-time PCR

Total RNA from cells and organizations were extracted with TRIzol reagent (Ambion, Life Technologies, CA, USA). The nuclear and cytosolic fractions from BMDMs were separated with the PARIS Kit (Thermo Scientific, Lithuania, USA) according to the instructions. The synthesis of cDNA was used RevertAid First Strand cDNA Synthesis Kit (ThermoFisher Scientific). Real-time PCR analysis was used QuantiNova SYBR-Green PCR kits (Qiagen, Germany) and performed on the CFX-96 (Bio-Rad, Hercules, CA, USA) according to the manufacturer’s instructions. The quantification of miRNA was used Bulge-Loop miRNA qRT-PCR Starter Kit (RiBoBio) according to the instructions. The relative level changes of mRNA, lncRNA or miRNA were calculated by the comparative CT (2^−ΔΔCT^) method. For expression of mRNA and lncRNA, *GAPDH* used as internal reference. For expression of miRNA, *U6* used as internal reference. All reactions were provided triplicate.

### Luciferase assay

For Luciferase assays, Promega Dual Luciferase Assay System (Promega, USA) was performed to confirm the direct binding ability of lncRNA AK089514/miR-125b-5p and miR-125b-5p/TRAF6. The wildtype (WT) 3-UTR and mutant sequence of TRAF6 containing miR-125b-5p binding sites were sub-cloned into the luciferase reporter vector psi-CHECK2 (Promega, Madison, WI, USA). Then cells were co-transfected with psi-CHECK2 vectors and miR-125b-5p mimic or miR-125b-5p inhibitor for 48 h. The chemiluminescence signals were detected and relative value (Luciferase/Renilla) was calculated following with the manufacturer's instruction. For lncRNA/miRNA interaction, WT and mutant sequence of lncRNA AK089514 were also sub-cloned into psi-CHECK2 luciferase reporter vectors, respectively. Then miR-125b-5p mimic or negative control (NC) mimic was transfected in cells pretreating with psi-CHECK2 vectors. The protocol of chemiluminescence signals measurement were similar. Each analysis was performed 3 times.

### Allergic asthma model and histopathology

To produce allergic asthma model, female C57BL/6 mice were sensitized by intraperitoneal injections of 100 μg Der f1 adsorbed to 2 mg of Imject Alum Adjuvant (Inject Alum; Thermo Scientific) in a volume of 200 μl PBS. Mice were sensitized by Der f1 or equal volume of PBS (negative control) at days 0, 7 and 14 of the experiment. From day 21 to 28, the final sensitization mice were challenged by aerosol exposure to either a Der f1 solution (50 μg Der f1 dissolved in 10 mL PBS) or PBS alone for 15 min every day. Following the last challenges, mice were sacrificed at day 29. For in vivo miR-125b-5p knockdown, miR-125b-5p antagomir (20 nmol in 40 μL saline), or control antagomir were administered intranasally on once a day from the day of the Der f1 challenge until sacrifice (days 21, 22, 23, 24, 25, 26 and 27). miR-125b-5p antagomir were designed and synthesized by RiBoBio Co. After euthanasia, the lungs were removed immediately, fixed with 10% formalin (phosphate-buffered) and embedded in paraffin. Following with deparaffinizing and hydrating, Sects. (5 μm) were prepared from paraffin-embedded lungs and stained with hematoxylin and eosin (H and E). Images were captured by a microscope (Eclipse 80i, Nikon, Japan). For quantitation of inflammatory scores, sections were examined by two blinded observers to determine the inflammatory infiltration of the lung.

### Flow analysis of cells from BALF

The methods of bronchoalveolar lavage fluid (BALF) from allergic asthma model was harvested with 0.8 mL ice-cold PBS as described previously [27]. For detection of macrophages, BALF was centrifuged at 1200 rpm for 5 min and the cell pellets were resuspended in an appropriate amount of PBS. Then cells were blocked by using the FcR Blocking Reagent (Miltenyi Biotech, Bergisch-Gladbach, Germany) for 20 min and then stained by using the anti-CD11c-PE-Cy7 (N418; Biolegend, San Diego, CA, USA) for 30 min at 4 °C. Following with fixation/permeabilization, cells were stained by using anti-iNOS-APC (CXNFT; Thermo Fisher Scientific, Waltham, MA, USA) or anti-CD206-APC (MR6F3; Thermo Fisher Scientific) for 30 min at 4 °C. The antibodies used above were either rat or rabbit antimouse. After washing once, the cells were analyzed using a CytoFLEX flow cytometer (Beckman, CA, USA).

### Western blot

Cells were lysed in laemmli (Sigma, USA). Protein concentration was assessed by bicinchoninic acid (BCA) assay. Total protein lysates were separate by Tricine-SDS-PAGE and transfer onto 0.22-μm nitrocellulose (NC) filter membranes (Millipore, Bedford, MA, USA). Antibodies used were anti TRAF6 (Abcam, MA, USA). β-actin was used as a control. Western blots were quantified by Image J software. All experiments were performed in triplicate, and the representative results were shown.

### Fluorescence in situ hybridization (FISH)

The FISH probe which specific targeting lncRNA AK089514 was designed and synthesized by RiboBio (Guangzhou, China). The hybridization performed in BMDMs had reported previously^28^. Images were captured with a confocal laser scanning microscope (Zeiss LSM800 confocal microscope, Mainz, Germany).

### RNA immunoprecipitation (RIP)

RIP assay was detected with a Magna RIP RNA binding Protein Immunoprecipitation Kit (Millipore, Bedford, MA, USA) according to the manufacturer's instructions. BMDM cells were harvest and treated with RIP lysis buffer. Then cell lysate was co-immunoprecipited with anti-Ago2 (Abcam) at 4 °C overnight. Anti-IgG was kept as a negative control. After elution, the immunoprecipitated RNAs were analyzed by RT-qPCR.

### Statistical analysis

Data are shown as the mean ± SD. Statistical analyses were employed with GraphPad Prism 5.0 (GraphPad Software, Inc., La Jolla, CA, USA). *P* < 0.05 was taken to indicate a statistically significant difference.

## Results

### miR-125b-5p is highly expressed in M(IL-4) polarized macrophages.

**Fig. 1:**
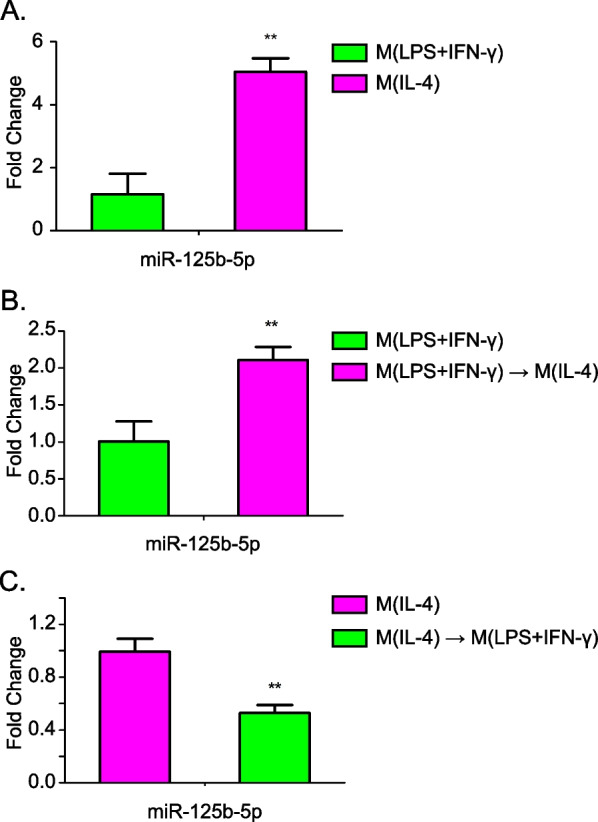
The expression level of miR-125b-5p during macrophage polarization. **A**. miR-125b-5p expression level was detected by RT-qPCR in BMDMs treated with LPS (100 ng/mL) plus IFN-γ (20 ng/mL) or IL-4 (20 ng/mL) for 48 h. **B**. miR-125b-5p expression level was detected in BMDMs macrophages following M(LPS + IFN-γ)-to-M(IL-4) re-polarization by IL-4 for 24 h. **C**. miR-125b-5p expression level was detected in BMDMs macrophages following M(IL-4)-to-M(LPS + IFN-γ) repolarization by LPS plus IFN-γ for 24 h. Data are mean ± SD from three independent experiments. **P < 0.01

An array assay was performed to determine the differently expressed miRNA between with M1 and M2 macrophages. Compared with M1 macrophages, we found that miR-125b-5p is one of the miRNAs that highly expressed in M2. Therefore, we further examined the role of miR-125b-5p in macrophage activation. Firstly, RT-PCR was used to confirm the miRNA array data. The expression of miR-125b-5p in M2 was higher than M1 (Fig. 1A). These data indicate that miR-125b-5p may be participated in the activation of the M2 macrophage phenotype.

Therefore, we further characterized miR-125b-5p role in macrophage polarization. The expression level of miR-125b-5p was assessed in macrophages after the dynamic process of macrophage re-polarization. Macrophages with the M(LPS + IFN-γ) phenotypes were re-polarized to the M(IL-4) phenotype by treatment with IL-4. Macrophages with the M(IL-4) phenotypes were re-polarized to the M(LPS + IFN-γ) phenotype by treatment with LPS/IFN-γ. The expression level of miR-125b-5p was increased following M(LPS + IFN-γ)-to-M(IL-4) re-polarization of macrophages, but decreased during M(IL-4)-to-M(LPS + IFN-γ) re-polarization of macrophages (Fig. 1B and C). These results indicate that miR-125b-5p may promote M(IL-4) polarization of macrophage.

### The role of miR-125b-5p in M(LPS + IFN-γ) and M(IL-4) macrophage polarization

**Fig. 2:**
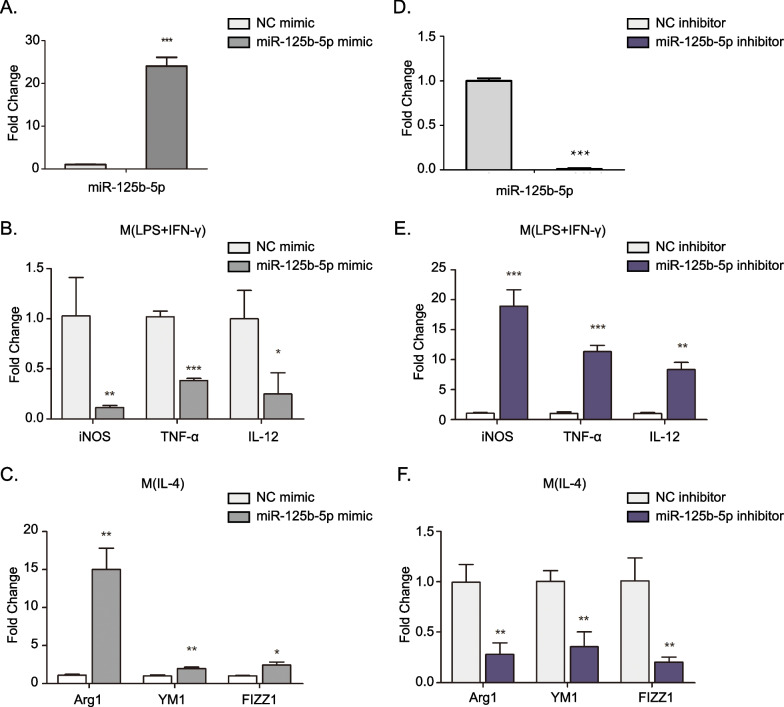
miR-125b-5p plays important roles in BMDMs polarization. BMDMs were transfected with NC or miR-125a-5p mimics for 48 h. **A** Relative expression of miR-125b-5p were detected by RT-PCR. **B** Transfected BMDMs were followed by stimulation with LPS + IFN-γ for an additional 24 h. Relative expression of iNOS, TNF-α, and IL-12 was detected. **C** Transfected BMDMs were followed by stimulation with IL-4 for an additional 24 h. Relative expression of Arg1, YM1, and FIZZ1 was detected. BMDMs were transfected with NC or miR-125a-5p inhibitor for 48 h. **D** Relative expression of miR-125b-5p were detected. **E** Transfected BMDMs were followed by stimulation with LPS + IFN-γ for an additional 24 h. Relative expression of iNOS, TNF-α, and IL-12 was detected. **F** Transfected BMDMs were followed by stimulation with IL-4 for an additional 24 h. Relative expression of Arg1, YM1, and FIZZ1 was detected. Data are mean ± SD from three independent experiments. *P < 0.05, **P < 0.01, ***P < 0.001

The expression profile of miR-125b-5p prompted us to investigate its function during macrophage polarization. To determine whether miR-125b-5p participates in macrophage polarization, miR-125b-5p was overexpressed or silenced in BMDM cells that treated with LPS/IFN-γ or IL-4. RT-qPCR results showed that miR-125b-5p expression was significant increase in primary BMDM cells which transfected with miR-125b-5p mimic (Fig. 2A). Upon elevation of the miR-125b-5p expression, the M(LPS + IFN-γ) macrophages stimulated by LPS and IFN-γ showed decreased expression of iNOS, TNF-α, and IL-12, While M(IL-4) macrophages induced by IL-4 showed increased expression of Arg1, YM1, and FIZZ1 (Fig. 2B and C). On the contrary, upon knockdown of the miR-125b-5p expression, the M(LPS + IFN-γ) macrophages stimulated by LPS and IFN-γ showed increased expression of iNOS, TNF-α, and IL-12, While M(IL-4) macrophages induced by IL-4 showed decreased expression of Arg1, YM1, and FIZZ1 (Fig. 2D–F). Taken together, these results indicated that miR-125b-5p may play an important role in promoting M2 macrophage polarization.

### Asthmatic mice exhibit abnormal miR-125b-5p expression.

**Fig. 3:**
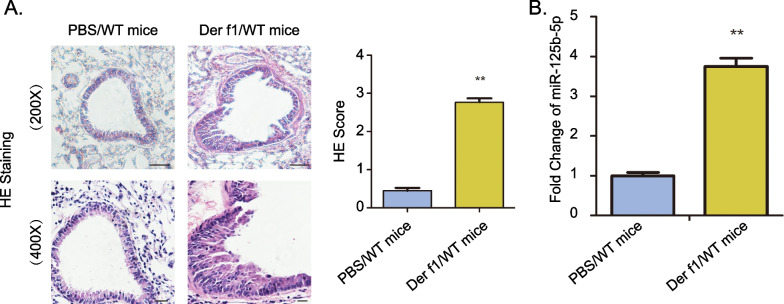
Asthmatic mice exhibit aberrant miR-125b-5p expression. **A** The histopathology of asthmatic inflammation in PBS or Der f1-stimulated mice were evaluated using H&E staining (magnification 400 × and 200 × , scale bar: 200 μm). **B** The expression of miR-125b-5p was detected in the BALF. Data are mean ± SD. n = 5 mice for each group. **P < 0.01

We previously confirmed that miR-125b-5p regulates the polarization of macrophages to M2. Recent studies have shown that M2 macrophages play critical roles in allergic asthma. Next, we investigated the role of miR-125b-5p in a mice model of M2 macrophage–associated, Der-f1-induced allergic asthma. Compared with PBS-challenged mice, the histologic examination of lung showed a notable accumulation of inflammatory cells and dense peribronchial infiltrates in Der-f1-challenged mice (Fig. 3A). Furthermore, the neutrophils, alveolar macrophages and eosinophils of BALF in Der f1-challenged WT mice were increased in compared to that of PBS-challenged WT mice (Additional file 1: Figure S1). This result indicate that asthma model was successfully constructed. Then we detected the expression of miR-125b-5p in the BALF cells of mice. The expression of miR-125b-5p was markedly higher in asthmatic mice than in healthy controls (Fig. 3B). Collectively, these data suggest that miR-125b-5p may involve in the development of allergic asthma.

### miR-125b-5p mice show impaired M2 macrophages polarization during allergic asthma

**Fig. 4:**
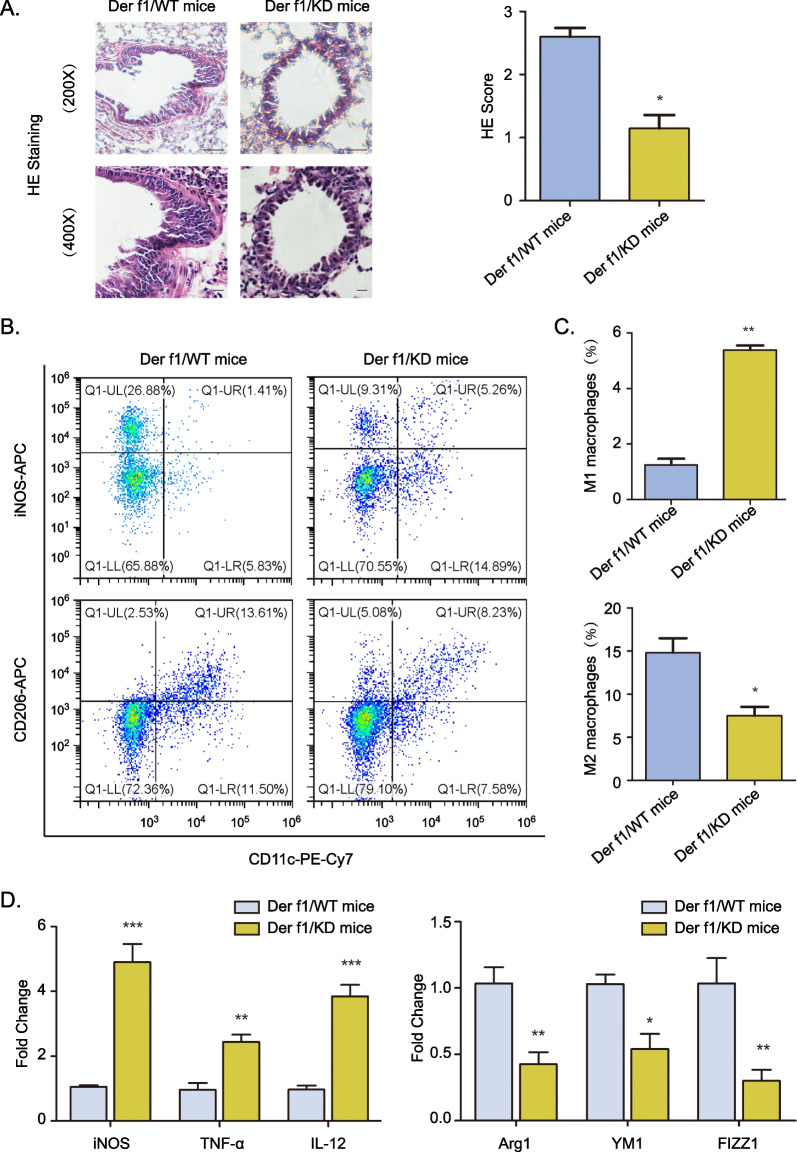
Knockdown of miR-125b-5p protects mice from the development of asthma via relieving M2 polarization in macrophages. **A** The histopathology of asthmatic inflammation in WT or KD mice stimulated by Der f1 were evaluated using H&E staining (magnification 400 × and 200 × , scale bar: 200 μm). **B** The CD206 and iNOS of macrophages from BALF of WT and miR-125b-5p KD mice which treatment with Der f1 was analyzed by flow cytometry. **C** Percentage of macrophages cells in B. **D** The expression levels of M1 associated genes and M2 associated genes from BALF were quantified using RT-PCR. Data are mean ± SD from three independent experiments. n = 5–6 mice for each group. *P < 0.05, **P < 0.01, ***P < 0.001

We next examined whether miR-125b-5p plays a role in mediating allergic inflammation. compared to Der f1-challenged WT, the histological results showing a notable accumulation of inflammatory cells to lungs with dense peribronchial infiltrates in miR-125b-5p KD mice (Fig. 4A). Then we investigated the macrophages of miR-125b-5p KD mice in asthma development. Flow cytometry indicated the number of M2 macrophages (CD206 marked) was decreased in the BALF of Der f1-challenged miR-125b-5p KD mice. Notably, M1 macrophages (iNOS marked) were increased in the BALF of miR-125b-5p KD mice (Fig. 4B and C). RT-PCR showed that the expression of the M2 marker, Arg1, YM1 and FIZZ1 were decreased in BALF cells from Der f1-challenged miR-125b-5p KD mice when compared with WT mice. miR-125b-5p KD result to increase the expression of the M1 marker, iNOS, TNF-α, and IL-12 (Fig. 4D). Together, these results suggest that miR-125b-5p deficiency slow down the Der f1-induced allergic inflammation through the inhibition of M2 macrophages activation.

### TRAF6 is a direct target of miR-125b-5p

**Fig. 5:**
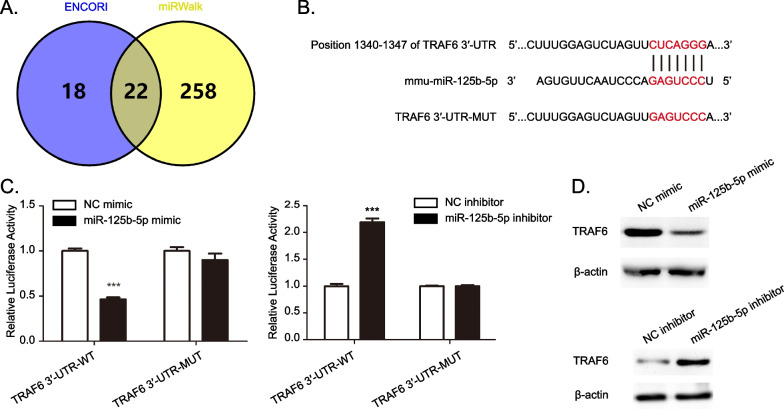
TRAF6 is a direct target gene of miR-125b-5p. **A** Venn diagram showing 22 putative miR-125b-5p target genes predicted by two databases (ENCORI and miRWalk). **B** Putative binding sequence between miR-125b-5p and the 3′-UTR region of TRAF6. **C** Dual luciferase reporter assay indicted that miR-125b-5p could bind to the 3′-UTR region of TRAF6. **D** Expression levels of TRAF6 were determined by western blot in miR-125b-5p-overexpressed or miR-125b-5p-silenced BMDMs cells. Data are mean ± SD from three independent experiments. ***P < 0.001

To determine the targets of miR-125b-5p, we performed bioinformatics analysis using two algorithms including ENCORI (The Encyclopedia of RNA Interactomes, https://starbase.sysu.edu.cn/agoClipRNA.php?source=mRNA) and miRWalk (http:// mirwalk.umm.uni-heidelberg.de/) databases. The prediction of the interactions of miRNA-targets were using the ENCORI which combining 5 target-predicting programs (PITA, miRmap, DIANA-microT, miRanda, PicTar) (Additional file 2: Table S1). The prediction of the interactions of miRNA-targets were using the miRWalk which combining selecting 2 target-predicting programs (TargetScan and miRDB) (Additional file 3: Table S2). As shows in Fig. 5A, 22 overlapped mRNAs were predicted the target genes of miR-125b-5p.

Among the miR-125b-5p targets that associated with inflammation, tumor necrosis factor receptor-associated factor (TRAF6) was of interest. TRAF6, a ubiquitin-conjugating enzyme that mediates nuclear factor κB (NF-κB) activation, are considered crucial orchestrators for inflammatory macrophage polarization [28]. TargetScan predicted that the binding sequence in the 3’-UTR of TRAF6 was complementary to the “seed site” of miR-125b-5p. We then mutated the miR-125b-5p binding site of the 3′-UTR of TRAF6 to construct the psi-CHECK2-TRAF6 3′-UTR-MUT vector (Fig. 5B). The luciferase reporter assay showed that miR-125b-5p overexpression inhibited luciferase activity of TRAF6 3’-UTR WT, but this repression was failure by mutation of the putative miR-125b-5p-binding site in the TRAF6 3’-UTR. On the contrary, miR-125b-5p KD promoted luciferase activity of TRAF6 3’-UTR WT (Fig. 5C). Next, miR-125b-5p mimics and miR-125b-5p inhibitor were transfected in the BMDMs to analyze the effect of the miR-125b-5p on TRAF6 expression. The over-expression of miR-125b-5p decreased the protein levels of TRAF6, but the miR-125b-5p inhibitor increased the TRAF6 expression (Fig. 5D and Additional file 1: Figure S2). Collectively, the above data indicate that miR-125b-5p regulates TRAF6 expression by directly binding to the predicted site in the 3′ UTR of TRAF6.

### miR-125b-5p targets lncRNA AK089514

**Fig. 6:**
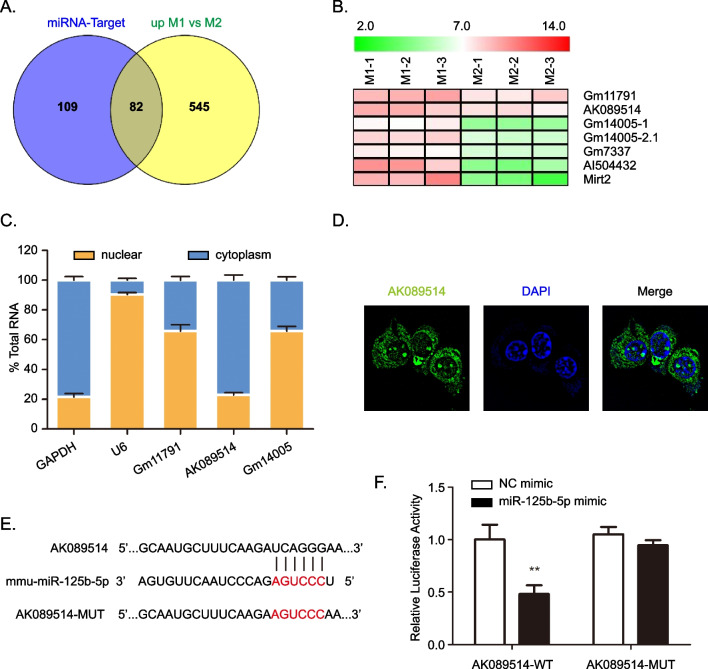
miR-125b-5p was the target of lncRNA AK089514. **A** Venn diagram showing 82 putative miR-125b-5p target lncRNAs predicted by TargetScan algorithms and upregulated in M1 macrophages. **B** The 7 key candidate lncRNAs was obtained by re-annotation. **C** RT-qPCR was used to detected the lncRNAs level in nuclear and cytoplasmic fractions from BMDMs. U6 and GAPDH were acted as the nuclear and cytoplasmic controls, respectively. **D** RNA FISH was used to detect the expression of lncRNA AK089514 in BMDMs. **E** Putative binding sequence between lncRNA AK089514 and miR-125b-5p. **F** Luciferase reporters containing WT or MUT lncRNA AK089514 transcript were co-transfected with miR-125b-5p mimics or negative control. Data are mean ± SD from three independent experiments. ** *P* < 0.01

To further explore the ceRNA mechanism of miR-125b-5p, we used Targetscan algorithm to predict 191 target lncRNAs. Then the 191 lncRNAs were crossed with the lncRNAs up-regulated in M1 macrophage from the lncRNA chip [29], 82 lncRNAs with trend were obtained (Fig. 6A and Additional file 4: Table S3). We obtain the 7 key candidate lncRNAs by reannotating the 82 lncRNAs (Fig. 6B). To detect the cellular localization of the candidate lncRNAs, the expressions of lncRNAs in nucleus and cytosol were measured. RT-PCR show that nearly 80% lncRNA AK089514 were expressed in the cytosol, most of lncRNA Gm11791 and Gm14005 were expressed in the nucleus (Fig. 6C). RNA FISH also indicated that lncRNA AK089514 is highly expressed in the cytosol (Fig. 6D). Furthermore, we constructed a mutant sequence of AK089514 that could not interact with miR-125b-5p (Fig. 6E). The luciferase reporter assays show miR-125b-5p mimics significantly inhibited the luciferase activity in cells transfected with the wildtype AK089514, whereas the luciferase activity was not obviously changed in cells transfected with the mutant AK089514 (Fig. 6F).

### The role of the AK089514/miR-125b-5p/TRAF6 axis in macrophages.

**Fig. 7:**
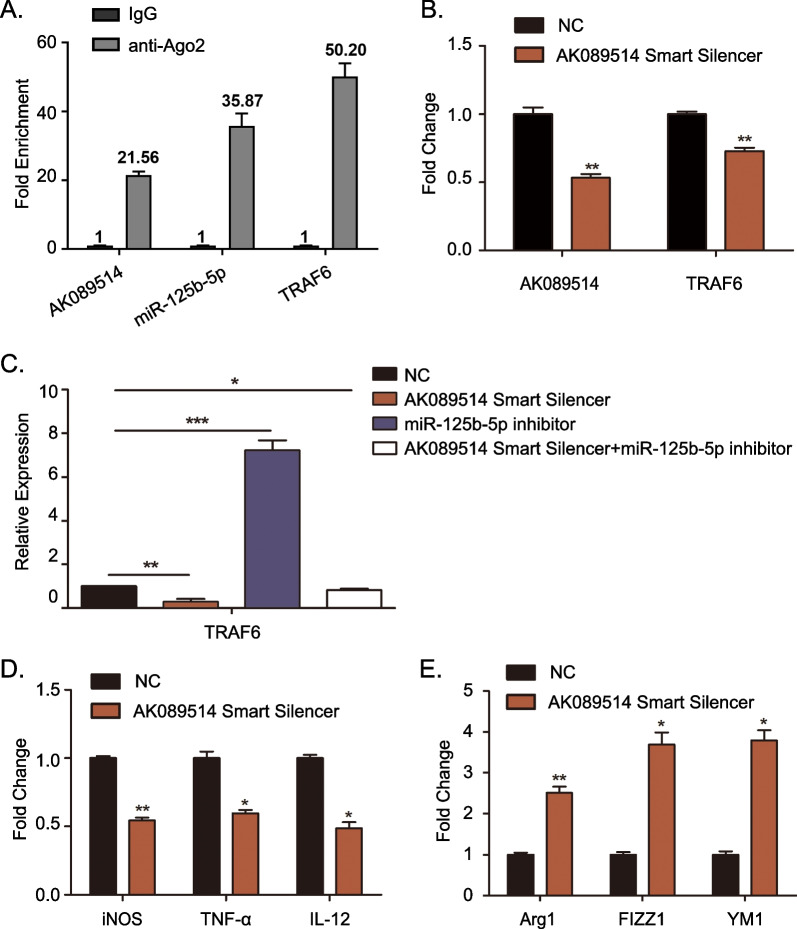
lncRNA AK089514 positively correlates with M1 macrophages in BMDMs cells. **A** RIP assay for Ago2 was employed to detect the levels of endogenous lncRNA AK089514, miR-125b-5p and TRAF6 in the Ago2 IP pellet. **B** Expression levels of lncRNA AK089514 and TRAF6 were detected by RT-PCR in lncRNA AK089514-silenced BMDMs cells. **C** Effects of miR-125b-5p underexpression on TRAF6 expression level in lncRNA AK089514-silenced BMDMs cells. **D** and **E** The expression levels of M1 associated genes and M2 associated genes from lncRNA AK089514-silenced BMDMs cells were quantified using RT-PCR

To confirm that TRAF6 is the target of miR-125b-5p regulated by AK089514, we performed a RIP assay using an anti-Ago2 antibody. RIP assay showed that endogenous lncRNA AK089514, miR-125b-5p and TRAF6 were enriched simultaneously with the anti-Ago2 compared to control IgG (Fig. 7A). We next detected whether AK089514 can regulate the expression of TRAF6. RT-PCR yielded that TRAF6 was downregulated in BMDM cells upon AK089514 knockdown (Fig. 7B). To explore whether miR-125b-5p plays a role in the relationship between AK089514 and TRAF6, BMDM cells were co-transfected with AK089514 smart silencer and miR-125b-5p inhibitor. Indeed, the expression levels of TRAF6 suppressed by AK089514 smart silencer was effectively reversed by the miR-125b-5p inhibitor (Fig. 7C). miR-125b-5p has been proved to be positive associated with M2 macrophage polarization. Next, we detected whether AK089514 could affect macrophage polarization in BMDM cells. Upon AK089514 smart silencer treatment, the M(LPS + IFN-γ) macrophages stimulated by LPS and IFN-γ showed decreased expression of iNOS, TNF-α, and IL-12, While M(IL-4) macrophages induced by IL-4 showed increased expression of Arg1, YM1, and FIZZ1 (Fig. 7D and E). Collectively, these data suggest that AK089514 involves in promoting macrophage polarization and modulates the expression of TRAF6 by post-transcriptional regulation of miR-125b-5p.

## Discussion

Asthma is a chronic inflammatory lung disease display uncontrolled airway inflammatory response [30]. M2 macrophages polarization plays a critical role in asthma by making airway inflammation more serious in asthma and inducing pulmonary inflammation [20]. MicroRNAs (miRNAs) are small endogenous non-coding RNAs (20–24 nucleotide-long) that function primarily as post-transcriptional regulators. More and more evidences have been shown miRNAs involves in the regulation of macrophage and subsequent effects on inflammation [31]. Accordingly, miRNA dysregulation could be related with asthma pathogenesis and therapeutic responsiveness to asthma.

We previously determined the expression profile of miRNAs in M1 and M2 macrophages (BMDM) with microarray assays. The chip data indicated that miR-125b-5p expression was significant increased during M1 to M2 polarization. In this study, we confirmed that miR-125b-5p is enriched in M2 macrophages and airway of asthma. MicroRNA-125 family (miR-125a and miR-125b) is a group of highly conserved microRNA. MiR-125b plays an important role in the immune system by regulating the expression of transcription factors, affecting cytokines and activating immune cells [32]. Here, we found that miR-125b-5p is decreased in M1 and functions as a negative regulator of macrophage-associated inflammatory responses. Consistent with previous studies which reported that miR-125b levels decrease in macrophages post-inflammatory stimulation for 3 h [33]. miR-125b has reported that targets TNF-α and involves in negative regulation of inflammatory responses [34–40]. We also found that miR-125b-5p KD effectively increased surface classical activation markers TNF-α, iNOS, IL-12 levels, supporting a physiological role for miR-125b-5p in macrophage polarization. Thus, miR-125b-5p may serve as a natural mechanism to anti-inflammatory responses.

In the competing endogenous RNAs (ceRNAs) network, lncRNAs and mRNAs acting as miRNA sponges to modulate the biological functions or expression of microRNAs [41]. In this study, bioinformatics analysis and luciferase reporter assays reveal that miR-125b-5p is a novel target of lncRNA AK089514. Furthermore, we confirmed that TRAF6 is one of the potential miR-125b-5p targets that have been reported. TRAF6 is a crucial intermediate in the NF-κB signaling pathway, and it can be activated by multiple stimuli, including DNA damage [42]. Consistent with our results, several reports showed that TRAF6 is a miR-125b-5p target gene. MicroRNA-125b attenuated cardiac dysfunction in polymicrobial sepsis through TRAF6-mediated nuclear factor κB (NF-κB) activation and p53-mediated apoptotic. miR-125b-5p targeting TRAF6 protectes skeletal muscle atrophy from fasting or denervation. Moreover, hsa-miR-125b-5p functions as a negative co-regulator of inflammatory genes by targeting TRAF6 in human osteoarthritic chondrocytes [43–45]. Here, we determined a mechanism of TRAF6 expression regulation that was modulated by noncoding RNA: miR-125b-5p repressed the expression of TRAF6, whereas AK089514 promoted the TRAF6 expression level by acting as a ceRNA to bind miR-125b-5p and relieve their repression on TRAF6.

Above all we show the miR-125b-5p acts as a checkpoint to inhibit abnormal activation of inflammation, and is a potential mediator of macrophage polarization. We identify a AK089514-miR-125b-5p-TRAF6 regulatory axis and clarify its function in macrophage polarization. Antagomir mediated gene transfer of miR-125b-5p protects mice from asthma. These findings identify miR-125b-5p as a negative feedback regulator of excessive inflammation. As such, this study identifies miR-125b-5p as a novel therapeutic target for treatment of asthma.

## Supplementary Information

**Additional file 1: Figure S1.** Cell distribution of BALF of in PBS or Der f1-treated mice; **Figure S2.** Full length blot for Figure 5D.

**Additional file 2: Table S1.** miR-125b-5p predicted targets from ENCORI.

**Additional file 3: Table S2.** miR-125b-5p predicted targets from miRWalk.

**Additional file 4: Table S3.** lncRNA target miR-125b-5p.

## Data Availability

All data generated or analysed during this study are included in this published article and its supplementary information files.

## Abbreviations

AA: Allergic asthma
BALF: Bronchoalveolar lavage fluid
Th2: T-helper type 2
BMDM: Bone marrow-derived macrophage
DMEM: Dulbecco’s modified Eagle’s medium
ncRNAs: Non-coding RNAs
Der f1: Dermatophagoides farinae protein
SPF: Specific-pathogen-free
FBS: Fetal bovine serum
FISH: Fluorescence in situ hybridization
lncRNA: Long non-coding RNA
NC: Nitrocellulose
BCA: Bicinchoninic acid
LPS: Lipopolysaccharide
M1: Classically activated macrophage
M2: Alternatively activated macrophage
KD: Knockdown
TRAF6: Tumor necrosis factor receptor-associated factor
ENCORI: The encyclopedia of RNA interactomes
RIP: RNA immunoprecipitation
H&E: Hematoxylin and eosin
ceRNA: Competing endogenous RNAs
