# Supplementary material for: LncRNA AK089514/miR-125b-5p/TRAF6 axis mediates macrophage polarization in allergic asthma
Source: BMC Pulm Med. 2023 Jan 30;23:45. doi: 10.1186/s12890-023-02339-1 (PMC9887860; doi:10.1186/s12890-023-02339-1)

Additional file 1:

**Figure S1. Cell distribution of BALF of in PBS or Der f1-treated mice.** (A) Gating strategy for BALF neutrophils (Neu: CD45^+^ Ly6G^+^). (B) Gating strategy for BALF alveolar macrophages (AM: CD45^+^ Ly6G^-^ CD11c^+^ Siglec F^+^) and eosinophils (Eos: CD45^+^ Ly6G^-^ CD11c^-^ Siglec F^+^). (C) Percentage of cells distribution in A and B. Data are mean ± SD from three independent experiments. **P < 0.01, *** P < 0.001.


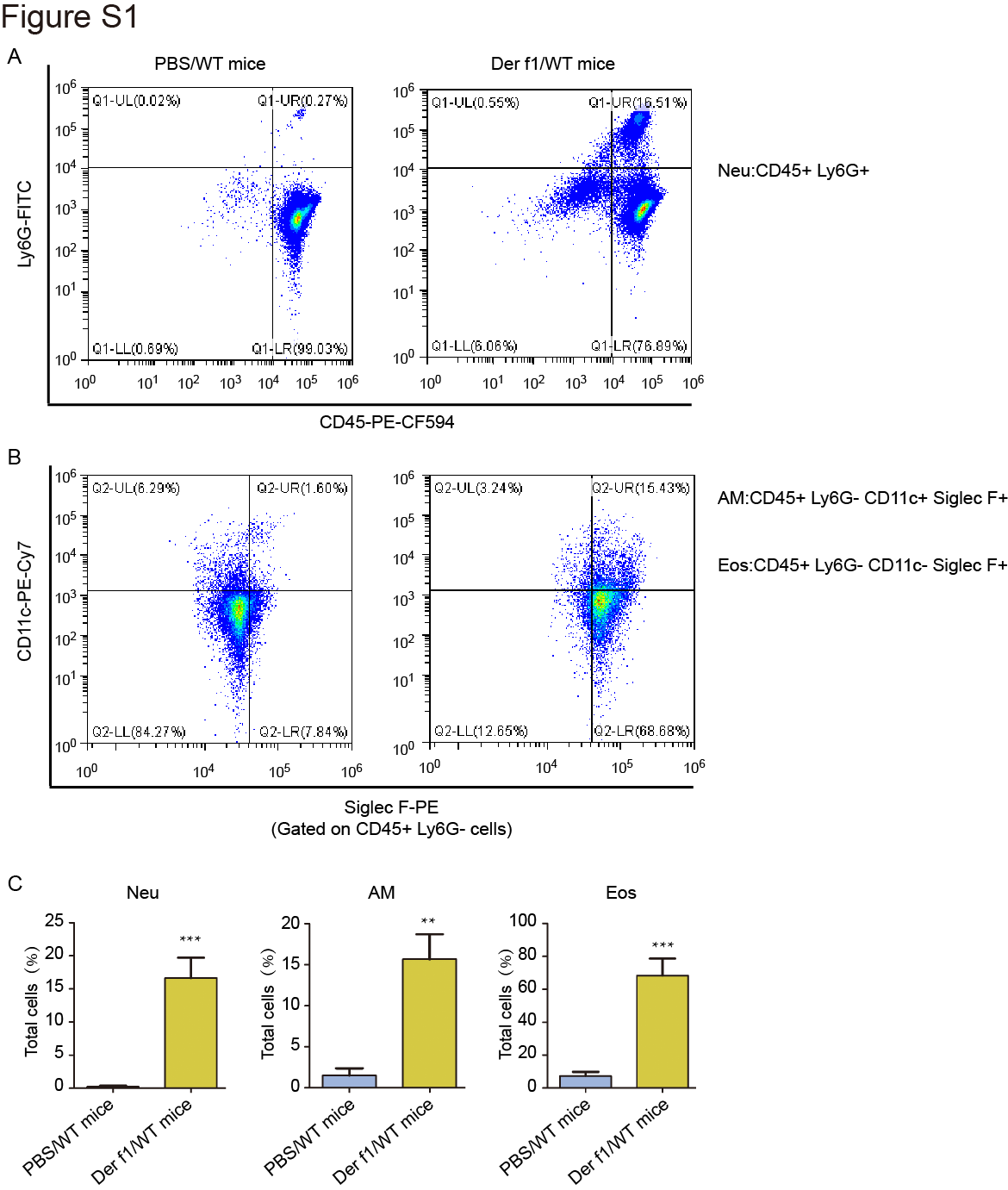


**Figure S2. Full length blot for Figure 5D.** (A) Expression levels of TRAF6 were determined by western blot in miR-125b-5p-overexpressed or miR-125b-5p-silenced BMDMs cells. This image is also the Figure 5D in the manuscript. (B) The original images of full-length blots for A（Figure 5D）.


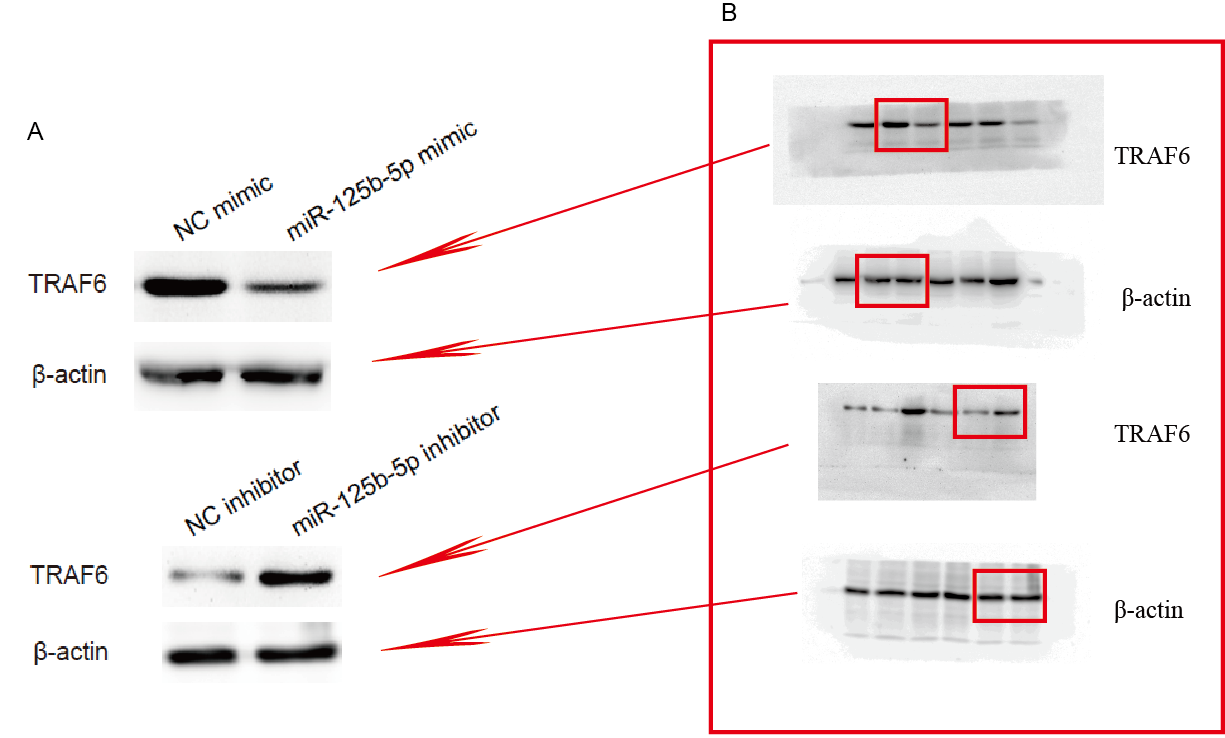

Supplement: Supplementary file 1 — Additional file 1: Figure S1. Cell distribution of BALF of in PBS or Der f1-treated mice; Figure S2. Full length blot for Figure 5D. [file 12890_2023_2339_MOESM1_ESM.docx]
