# Supplementary material for: LncRNA AK089514/miR-125b-5p/TRAF6 axis mediates macrophage polarization in allergic asthma
Source: BMC Pulm Med. 2023 Jan 30;23:45. doi: 10.1186/s12890-023-02339-1 (PMC9887860; doi:10.1186/s12890-023-02339-1)
Supplement: Supplementary file 2 — Additional file 2: Table S1. miR-125b-5p predicted targets from ENCORI. [file 12890_2023_2339_MOESM2_ESM.pdf]

Table S1. miR-125b-5p predicted targets from ENCORI

miRNAname	geneName	clipExpNum	RBP	PITA	miRmap	microT	miRanda	PicTar
mmu-miR-125b-5p	Cpsf6	6	Ago2	1	1	1	1	1
mmu-miR-125b-5p	Rhoq	6	Ago1-4,Ago2	1	1	1	1	1
mmu-miR-125b-5p	Lfng	6	Ago1-4,Ago2	1	1	1	1	1
mmu-miR-125b-5p	Rab8b	6	Ago1-4,Ago2	1	1	1	1	1
mmu-miR-125b-5p	Vps4b	5	Ago2	1	1	1	1	1
mmu-miR-125b-5p	Sema4d	5	Ago1-4,Ago2	1	1	1	1	1
mmu-miR-125b-5p	Khnyn	5	Ago2	1	1	1	1	1
mmu-miR-125b-5p	Dram2	5	Ago1-4,Ago2	1	1	1	1	1
mmu-miR-125b-5p	Blzf1	4	Ago2	1	1	1	1	1
mmu-miR-125b-5p	Ppp4r3a	4	Ago1-4,Ago2	1	1	1	1	1
mmu-miR-125b-5p	Rreb1	4	Ago1-4,Ago2	1	1	1	1	1
mmu-miR-125b-5p	Lnpep	4	Ago2	1	1	1	1	1
mmu-miR-125b-5p	Cdc37l1	4	Ago2	1	1	1	1	1
mmu-miR-125b-5p	Ptpn1	4	Ago2	1	1	1	1	1
mmu-miR-125b-5p	Mcl1	4	Ago1-4,Ago2	1	1	1	1	1
mmu-miR-125b-5p	Triap1	4	Ago1-4,Ago2	1	1	1	1	1
mmu-miR-125b-5p	Nup210	4	Ago1-4,Ago2	1	1	1	1	1
mmu-miR-125b-5p	Ist1	4	Ago1-4,Ago2	1	1	1	1	1
mmu-miR-125b-5p	Lactb	4	Ago1-4,Ago2	1	1	1	1	1
mmu-miR-125b-5p	Trim71	4	Ago2	1	1	1	1	1
mmu-miR-125b-5p	Ptpn18	3	Ago2	1	1	1	1	1
mmu-miR-125b-5p	Prdm1	3	Ago2	1	1	1	1	1
mmu-miR-125b-5p	Sgpl1	3	Ago2	1	1	1	1	1
mmu-miR-125b-5p	Pctp	3	Ago2	1	1	1	1	1
mmu-miR-125b-5p	Gjc1	3	Ago1-4,Ago2	1	1	1	1	1
mmu-miR-125b-5p	Zfyve1	3	Ago2	1	1	1	1	1
mmu-miR-125b-5p	Ppp2r5c	3	Ago2	1	1	1	1	1
mmu-miR-125b-5p	Fam118a	3	Ago1-4,Ago2	1	1	1	1	1
mmu-miR-125b-5p	Atl2	3	Ago1-4,Ago2	1	1	1	1	1
mmu-miR-125b-5p	Apc	3	Ago1-4,Ago2	1	1	1	1	1
mmu-miR-125b-5p	Hnrnpul2	3	Ago2	1	1	1	1	1
mmu-miR-125b-5p	Zfp518a	3	Ago2	1	1	1	1	1
mmu-miR-125b-5p	Bmf	3	Ago2	1	1	1	1	1
mmu-miR-125b-5p	Traf6	3	Ago2	1	1	1	1	1
mmu-miR-125b-5p	Ptpn1	3	Ago2	1	1	1	1	1
mmu-miR-125b-5p	Osbpl9	3	Ago1-4,Ago2	1	1	1	1	1
mmu-miR-125b-5p	Trp53inp1	3	Ago2	1	1	1	1	1
mmu-miR-125b-5p	Sema4b	3	Ago2	1	1	1	1	1
mmu-miR-125b-5p	Galnt7	3	Ago2	1	1	1	1	1
mmu-miR-125b-5p	Mfhas1	3	Ago2	1	1	1	1	1
mmu-miR-125b-5p	Fmr1	3	Ago2	1	1	1	1	1
