# Supplementary material for: LncRNA AK089514/miR-125b-5p/TRAF6 axis mediates macrophage polarization in allergic asthma
Source: BMC Pulm Med. 2023 Jan 30;23:45. doi: 10.1186/s12890-023-02339-1 (PMC9887860; doi:10.1186/s12890-023-02339-1)
Supplement: Supplementary file 3 — Additional file 3: Table S2. miR-125b-5p predicted targets from miRWalk. [file 12890_2023_2339_MOESM3_ESM.pdf]

Table S2. miR-125b-5p predicted targets from miRWalk

miRNA ID	genesymbol	position	TargetScan	miRDB
mmu-miR-125b-5p	Trps1	3UTR	1	1
mmu-miR-125b-5p	Fam126b	3UTR	1	1
mmu-miR-125b-5p	Fam126b	3UTR	1	1
mmu-miR-125b-5p	Fam126b	3UTR	1	1
mmu-miR-125b-5p	Tbc1d1	3UTR	1	1
mmu-miR-125b-5p	Glb1l2	3UTR	1	1
mmu-miR-125b-5p	Bmf	3UTR	1	1
mmu-miR-125b-5p	Bmf	3UTR	1	1
mmu-miR-125b-5p	Kcnk10	3UTR	1	1
mmu-miR-125b-5p	Kcnk10	3UTR	1	1
mmu-miR-125b-5p	Sgpl1	3UTR	1	1
mmu-miR-125b-5p	Itga7	3UTR	1	1
mmu-miR-125b-5p	Abhd6	3UTR	1	1
mmu-miR-125b-5p	Abhd6	3UTR	1	1
mmu-miR-125b-5p	Bmf	3UTR	1	1
mmu-miR-125b-5p	Bmf	3UTR	1	1
mmu-miR-125b-5p	Osbpl9	3UTR	1	1
mmu-miR-125b-5p	Elavl4	3UTR	1	1
mmu-miR-125b-5p	Slc8a2	3UTR	1	1
mmu-miR-125b-5p	Tbc1d16	3UTR	1	1
mmu-miR-125b-5p	Tbc1d16	3UTR	1	1
mmu-miR-125b-5p	Fgfr2	3UTR	1	1
mmu-miR-125b-5p	Rfx5	3UTR	1	1
mmu-miR-125b-5p	Dvl1	3UTR	1	1
mmu-miR-125b-5p	Zfp385a	3UTR	1	1
mmu-miR-125b-5p	Fndc3b	3UTR	1	1
mmu-miR-125b-5p	Psmg3	3UTR	1	1
mmu-miR-125b-5p	Slc46a3	3UTR	1	1
mmu-miR-125b-5p	Tmem25	3UTR	1	1
mmu-miR-125b-5p	Zfp687	3UTR	1	1
mmu-miR-125b-5p	Zfp523	3UTR	1	1
mmu-miR-125b-5p	Lifr	3UTR	1	1
mmu-miR-125b-5p	Zfyve1	3UTR	1	1
mmu-miR-125b-5p	Zfyve1	3UTR	1	1
mmu-miR-125b-5p	Zfyve1	3UTR	1	1
mmu-miR-125b-5p	Sbno1	3UTR	1	1
mmu-miR-125b-5p	Sbno1	3UTR	1	1
mmu-miR-125b-5p	Sbno1	3UTR	1	1
mmu-miR-125b-5p	Sbno1	3UTR	1	1
mmu-miR-125b-5p	E2f3	3UTR	1	1
mmu-miR-125b-5p	Kctd15	3UTR	1	1
mmu-miR-125b-5p	Kctd15	3UTR	1	1
mmu-miR-125b-5p	B3galnt2	3UTR	1	1
mmu-miR-125b-5p	Chtf8	3UTR	1	1
mmu-miR-125b-5p	Ppp2r5c	3UTR	1	1
mmu-miR-125b-5p	Ppp2r5c	3UTR	1	1
mmu-miR-125b-5p	Osbpl9	3UTR	1	1
mmu-miR-125b-5p	Rusc2	3UTR	1	1
mmu-miR-125b-5p	Acads	3UTR	1	1
mmu-miR-125b-5p	Bak1	3UTR	1	1

mmu-miR-125b-5p	Bak1	3UTR	1	1
mmu-miR-125b-5p	Bcl2l2	3UTR	1	1
mmu-miR-125b-5p	Prdm1	3UTR	1	1
mmu-miR-125b-5p	Bsn	3UTR	1	1
mmu-miR-125b-5p	Btg2	3UTR	1	1
mmu-miR-125b-5p	Cacna1b	3UTR	1	1
mmu-miR-125b-5p	Cacna1b	3UTR	1	1
mmu-miR-125b-5p	Casp2	3UTR	1	1
mmu-miR-125b-5p	Csnk2a1	3UTR	1	1
mmu-miR-125b-5p	Csnk2a1	3UTR	1	1
mmu-miR-125b-5p	Arid3a	3UTR	1	1
mmu-miR-125b-5p	Fut1	3UTR	1	1
mmu-miR-125b-5p	Fut1	3UTR	1	1
mmu-miR-125b-5p	Itga7	3UTR	1	1
mmu-miR-125b-5p	Anpep	3UTR	1	1
mmu-miR-125b-5p	Lep	3UTR	1	1
mmu-miR-125b-5p	Lfng	3UTR	1	1
mmu-miR-125b-5p	Lif	3UTR	1	1
mmu-miR-125b-5p	Lif	3UTR	1	1
mmu-miR-125b-5p	Mcl1	3UTR	1	1
mmu-miR-125b-5p	Mtf1	3UTR	1	1
mmu-miR-125b-5p	Myt1	3UTR	1	1
mmu-miR-125b-5p	Myt1	3UTR	1	1
mmu-miR-125b-5p	Nfib	3UTR	1	1
mmu-miR-125b-5p	Pctp	3UTR	1	1
mmu-miR-125b-5p	Pctp	3UTR	1	1
mmu-miR-125b-5p	Pik3ca	3UTR	1	1
mmu-miR-125b-5p	Plxna1	3UTR	1	1
mmu-miR-125b-5p	Sgpl1	3UTR	1	1
mmu-miR-125b-5p	St8sia4	3UTR	1	1
mmu-miR-125b-5p	St8sia4	3UTR	1	1
mmu-miR-125b-5p	Sstr3	3UTR	1	1
mmu-miR-125b-5p	Sox11	3UTR	1	1
mmu-miR-125b-5p	Sox11	3UTR	1	1
mmu-miR-125b-5p	Stc1	3UTR	1	1
mmu-miR-125b-5p	Tle3	3UTR	1	1
mmu-miR-125b-5p	Tnfaip3	3UTR	1	1
mmu-miR-125b-5p	Traf6	3UTR	1	1
mmu-miR-125b-5p	Ube2l3	3UTR	1	1
mmu-miR-125b-5p	Unc5c	3UTR	1	1
mmu-miR-125b-5p	Wnt11	3UTR	1	1
mmu-miR-125b-5p	Wnt11	3UTR	1	1
mmu-miR-125b-5p	Wnt7a	3UTR	1	1
mmu-miR-125b-5p	Wnt7a	3UTR	1	1
mmu-miR-125b-5p	Plagl1	3UTR	1	1
mmu-miR-125b-5p	Asic1	3UTR	1	1
mmu-miR-125b-5p	Cyp24a1	3UTR	1	1
mmu-miR-125b-5p	Cyp24a1	3UTR	1	1
mmu-miR-125b-5p	Dvl1	3UTR	1	1
mmu-miR-125b-5p	Edn1	3UTR	1	1
mmu-miR-125b-5p	Fgfr2	3UTR	1	1
mmu-miR-125b-5p	Fmn1	3UTR	1	1
mmu-miR-125b-5p	Gab2	3UTR	1	1
mmu-miR-125b-5p	Gab2	3UTR	1	1

mmu-miR-125b-5p	Nr6a1	3UTR	1	1
mmu-miR-125b-5p	Nr6a1	3UTR	1	1
mmu-miR-125b-5p	Gcnt1	3UTR	1	1
mmu-miR-125b-5p	Gcnt1	3UTR	1	1
mmu-miR-125b-5p	Grb10	3UTR	1	1
mmu-miR-125b-5p	Hoxb3	3UTR	1	1
mmu-miR-125b-5p	Hoxb3	3UTR	1	1
mmu-miR-125b-5p	Elavl4	3UTR	1	1
mmu-miR-125b-5p	Ajuba	3UTR	1	1
mmu-miR-125b-5p	Kcnh1	3UTR	1	1
mmu-miR-125b-5p	Loxl1	3UTR	1	1
mmu-miR-125b-5p	Loxl1	3UTR	1	1
mmu-miR-125b-5p	Numbl	3UTR	1	1
mmu-miR-125b-5p	Rfx3	3UTR	1	1
mmu-miR-125b-5p	Rfxank	3UTR	1	1
mmu-miR-125b-5p	Sim1	3UTR	1	1
mmu-miR-125b-5p	Sim1	3UTR	1	1
mmu-miR-125b-5p	Sim1	3UTR	1	1
mmu-miR-125b-5p	Suv39h1	3UTR	1	1
mmu-miR-125b-5p	Tbx4	3UTR	1	1
mmu-miR-125b-5p	Tdg	3UTR	1	1
mmu-miR-125b-5p	Tnfrsf1b	3UTR	1	1
mmu-miR-125b-5p	Wars	3UTR	1	1
mmu-miR-125b-5p	Ikzf3	3UTR	1	1
mmu-miR-125b-5p	Ikzf3	3UTR	1	1
mmu-miR-125b-5p	Ikzf4	3UTR	1	1
mmu-miR-125b-5p	Ets1	3UTR	1	1
mmu-miR-125b-5p	Map2k7	3UTR	1	1
mmu-miR-125b-5p	Ppp2r5c	3UTR	1	1
mmu-miR-125b-5p	Ppp2r5c	3UTR	1	1
mmu-miR-125b-5p	Hapln1	3UTR	1	1
mmu-miR-125b-5p	Lifr	3UTR	1	1
mmu-miR-125b-5p	Rora	3UTR	1	1
mmu-miR-125b-5p	Golga5	3UTR	1	1
mmu-miR-125b-5p	Zfp385a	3UTR	1	1
mmu-miR-125b-5p	Sult4a1	3UTR	1	1
mmu-miR-125b-5p	Fbxw4	3UTR	1	1
mmu-miR-125b-5p	Hnf4g	3UTR	1	1
mmu-miR-125b-5p	Usp2	3UTR	1	1
mmu-miR-125b-5p	St6galnac6	3UTR	1	1
mmu-miR-125b-5p	St6galnac6	3UTR	1	1
mmu-miR-125b-5p	St6galnac6	3UTR	1	1
mmu-miR-125b-5p	Rfx5	3UTR	1	1
mmu-miR-125b-5p	Dnal4	3UTR	1	1
mmu-miR-125b-5p	Nup210	3UTR	1	1
mmu-miR-125b-5p	Sfmbt1	3UTR	1	1
mmu-miR-125b-5p	Sfmbt1	3UTR	1	1
mmu-miR-125b-5p	Tbc1d1	3UTR	1	1
mmu-miR-125b-5p	Arid3b	3UTR	1	1
mmu-miR-125b-5p	Rybp	3UTR	1	1
mmu-miR-125b-5p	Kcnip3	3UTR	1	1
mmu-miR-125b-5p	Kcnip3	3UTR	1	1
mmu-miR-125b-5p	Srf	3UTR	1	1
mmu-miR-125b-5p	Srf	3UTR	1	1

mmu-miR-125b-5p	Klf13	3UTR	1	1
mmu-miR-125b-5p	Srrm3	3UTR	1	1
mmu-miR-125b-5p	Necab3	3UTR	1	1
mmu-miR-125b-5p	Trp53inp1	3UTR	1	1
mmu-miR-125b-5p	Bhmt2	3UTR	1	1
mmu-miR-125b-5p	Rmnd5a	3UTR	1	1
mmu-miR-125b-5p	Abhd6	3UTR	1	1
mmu-miR-125b-5p	Blzf1	3UTR	1	1
mmu-miR-125b-5p	Psmg3	3UTR	1	1
mmu-miR-125b-5p	Ubr7	3UTR	1	1
mmu-miR-125b-5p	Ube2w	3UTR	1	1
mmu-miR-125b-5p	Tril	3UTR	1	1
mmu-miR-125b-5p	Med28	3UTR	1	1
mmu-miR-125b-5p	Cdc37l1	3UTR	1	1
mmu-miR-125b-5p	Pi4k2b	3UTR	1	1
mmu-miR-125b-5p	Dynlt3	3UTR	1	1
mmu-miR-125b-5p	Ube2g1	3UTR	1	1
mmu-miR-125b-5p	Dram2	3UTR	1	1
mmu-miR-125b-5p	Daam1	3UTR	1	1
mmu-miR-125b-5p	Ube2r2	3UTR	1	1
mmu-miR-125b-5p	Ube2r2	3UTR	1	1
mmu-miR-125b-5p	Slc35a4	3UTR	1	1
mmu-miR-125b-5p	Atp5g2	3UTR	1	1
mmu-miR-125b-5p	Retreg3	3UTR	1	1
mmu-miR-125b-5p	Retreg3	3UTR	1	1
mmu-miR-125b-5p	Taf9b	3UTR	1	1
mmu-miR-125b-5p	Itga8	3UTR	1	1
mmu-miR-125b-5p	Mtus1	3UTR	1	1
mmu-miR-125b-5p	Mtus1	3UTR	1	1
mmu-miR-125b-5p	Mtus1	3UTR	1	1
mmu-miR-125b-5p	Mtus1	3UTR	1	1
mmu-miR-125b-5p	Mtus1	3UTR	1	1
mmu-miR-125b-5p	Mtus1	3UTR	1	1
mmu-miR-125b-5p	Zbtb9	3UTR	1	1
mmu-miR-125b-5p	Phactr3	3UTR	1	1
mmu-miR-125b-5p	Scn4b	3UTR	1	1
mmu-miR-125b-5p	Scn2b	3UTR	1	1
mmu-miR-125b-5p	Scn2b	3UTR	1	1
mmu-miR-125b-5p	St6galnac6	3UTR	1	1
mmu-miR-125b-5p	St6galnac6	3UTR	1	1
mmu-miR-125b-5p	St6galnac6	3UTR	1	1
mmu-miR-125b-5p	St6galnac6	3UTR	1	1
mmu-miR-125b-5p	St6galnac6	3UTR	1	1
mmu-miR-125b-5p	St6galnac6	3UTR	1	1
mmu-miR-125b-5p	Dram2	3UTR	1	1
mmu-miR-125b-5p	Rfxank	3UTR	1	1
mmu-miR-125b-5p	Zswim5	3UTR	1	1
mmu-miR-125b-5p	Zswim5	3UTR	1	1
mmu-miR-125b-5p	Man1b1	3UTR	1	1
mmu-miR-125b-5p	Man1b1	3UTR	1	1
mmu-miR-125b-5p	Lin28b	3UTR	1	1
mmu-miR-125b-5p	Smg1	3UTR	1	1
mmu-miR-125b-5p	Cln6	3UTR	1	1
mmu-miR-125b-5p	C77080	3UTR	1	1

mmu-miR-125b-5p	C77080	3UTR	1	1
mmu-miR-125b-5p	Tlcd5	3UTR	1	1
mmu-miR-125b-5p	Rusc2	3UTR	1	1
mmu-miR-125b-5p	Kcnh1	3UTR	1	1
mmu-miR-125b-5p	Smurf1	3UTR	1	1
mmu-miR-125b-5p	Smurf1	3UTR	1	1
mmu-miR-125b-5p	Ets1	3UTR	1	1
mmu-miR-125b-5p	Elavl4	3UTR	1	1
mmu-miR-125b-5p	Kctd21	3UTR	1	1
mmu-miR-125b-5p	Plekhm3	3UTR	1	1
mmu-miR-125b-5p	Plekhm3	3UTR	1	1
mmu-miR-125b-5p	Lif	3UTR	1	1
mmu-miR-125b-5p	Lif	3UTR	1	1
mmu-miR-125b-5p	Tmem120b	3UTR	1	1
mmu-miR-125b-5p	Cacna1b	3UTR	1	1
mmu-miR-125b-5p	Cacna1b	3UTR	1	1
mmu-miR-125b-5p	Rorb	3UTR	1	1
mmu-miR-125b-5p	Ccnjl	3UTR	1	1
mmu-miR-125b-5p	Slc1a2	3UTR	1	1
mmu-miR-125b-5p	Slc1a2	3UTR	1	1
mmu-miR-125b-5p	Slc1a2	3UTR	1	1
mmu-miR-125b-5p	Slc1a2	3UTR	1	1
mmu-miR-125b-5p	Slc1a2	3UTR	1	1
mmu-miR-125b-5p	Hoxb3	3UTR	1	1
mmu-miR-125b-5p	Hoxb3	3UTR	1	1
mmu-miR-125b-5p	Uhrf1bp1	3UTR	1	1
mmu-miR-125b-5p	Rapgefl1	3UTR	1	1
mmu-miR-125b-5p	Rapgefl1	3UTR	1	1
mmu-miR-125b-5p	Lclat1	3UTR	1	1
mmu-miR-125b-5p	Hnrnpul2	3UTR	1	1
mmu-miR-125b-5p	Hnrnpul2	3UTR	1	1
mmu-miR-125b-5p	Hnrnpul2	3UTR	1	1
mmu-miR-125b-5p	Sbno1	3UTR	1	1
mmu-miR-125b-5p	Sbno1	3UTR	1	1
mmu-miR-125b-5p	Alg6	3UTR	1	1
mmu-miR-125b-5p	Mfhas1	3UTR	1	1
mmu-miR-125b-5p	Ino80d	3UTR	1	1
mmu-miR-125b-5p	Ppp2r5c	3UTR	1	1
mmu-miR-125b-5p	Ppp2r5c	3UTR	1	1
mmu-miR-125b-5p	Ppp2r5c	3UTR	1	1
mmu-miR-125b-5p	Tbc1d8b	3UTR	1	1
mmu-miR-125b-5p	Tbc1d8b	3UTR	1	1
mmu-miR-125b-5p	Slc35a4	3UTR	1	1
mmu-miR-125b-5p	Greb1l	3UTR	1	1
mmu-miR-125b-5p	Tle3	3UTR	1	1
mmu-miR-125b-5p	Tle3	3UTR	1	1
mmu-miR-125b-5p	Zbtb34	3UTR	1	1
mmu-miR-125b-5p	Cd34	3UTR	1	1
mmu-miR-125b-5p	Lrrc10b	3UTR	1	1
mmu-miR-125b-5p	Kcnip3	3UTR	1	1
mmu-miR-125b-5p	Nfib	3UTR	1	1
mmu-miR-125b-5p	Nfib	3UTR	1	1
mmu-miR-125b-5p	Fam131b	3UTR	1	1
mmu-miR-125b-5p	Itga9	3UTR	1	1

mmu-miR-125b-5p	Slc25a53	3UTR	1	1
mmu-miR-125b-5p	Ino80d	3UTR	1	1
mmu-miR-125b-5p	Stxbp5l	3UTR	1	1
mmu-miR-125b-5p	Stxbp5l	3UTR	1	1
mmu-miR-125b-5p	Stxbp5l	3UTR	1	1
mmu-miR-125b-5p	Stxbp5l	3UTR	1	1
mmu-miR-125b-5p	Gcnt1	3UTR	1	1
mmu-miR-125b-5p	Gcnt1	3UTR	1	1
mmu-miR-125b-5p	Rnf44	3UTR	1	1
mmu-miR-125b-5p	Rnf44	3UTR	1	1
mmu-miR-125b-5p	Nr6a1	3UTR	1	1
mmu-miR-125b-5p	Nr6a1	3UTR	1	1
mmu-miR-125b-5p	Nr6a1	3UTR	1	1
mmu-miR-125b-5p	Ppp1r16b	3UTR	1	1
mmu-miR-125b-5p	Blzf1	3UTR	1	1
mmu-miR-125b-5p	Blzf1	3UTR	1	1
mmu-miR-125b-5p	Ppp4r3a	3UTR	1	1
mmu-miR-125b-5p	Fam78b	3UTR	1	1
mmu-miR-125b-5p	Fam78b	3UTR	1	1
mmu-miR-125b-5p	Fam78b	3UTR	1	1
mmu-miR-125b-5p	Fam78b	3UTR	1	1
mmu-miR-125b-5p	Tent5a	3UTR	1	1
mmu-miR-125b-5p	Tent5a	3UTR	1	1
mmu-miR-125b-5p	Tent5a	3UTR	1	1
mmu-miR-125b-5p	Tent5a	3UTR	1	1
mmu-miR-125b-5p	Gab2	3UTR	1	1
mmu-miR-125b-5p	Gab2	3UTR	1	1
mmu-miR-125b-5p	Mapre2	3UTR	1	1
mmu-miR-125b-5p	Mapre2	3UTR	1	1
mmu-miR-125b-5p	Elavl4	3UTR	1	1
mmu-miR-125b-5p	Elavl4	3UTR	1	1
mmu-miR-125b-5p	Crb2	3UTR	1	1
mmu-miR-125b-5p	Ppp6r3	3UTR	1	1
mmu-miR-125b-5p	Map2k7	3UTR	1	1
mmu-miR-125b-5p	Wars	3UTR	1	1
mmu-miR-125b-5p	Ankrd33b	3UTR	1	1
mmu-miR-125b-5p	Ankrd33b	3UTR	1	1
mmu-miR-125b-5p	Ankrd33b	3UTR	1	1
mmu-miR-125b-5p	Wars	3UTR	1	1
mmu-miR-125b-5p	Coro2a	3UTR	1	1
mmu-miR-125b-5p	Tnfaip3	3UTR	1	1
mmu-miR-125b-5p	Galnt7	3UTR	1	1
mmu-miR-125b-5p	Taf9b	3UTR	1	1
mmu-miR-125b-5p	Taf9b	3UTR	1	1
mmu-miR-125b-5p	Fam83h	3UTR	1	1
mmu-miR-125b-5p	Mlf2	3UTR	1	1
mmu-miR-125b-5p	Mlf2	3UTR	1	1
mmu-miR-125b-5p	Rbm20	3UTR	1	1
mmu-miR-125b-5p	Myt1	3UTR	1	1
mmu-miR-125b-5p	Myt1	3UTR	1	1
mmu-miR-125b-5p	Myt1	3UTR	1	1
mmu-miR-125b-5p	Myt1	3UTR	1	1
mmu-miR-125b-5p	Myt1	3UTR	1	1
mmu-miR-125b-5p	Myt1	3UTR	1	1

mmu-miR-125b-5p	Dis3l2	3UTR	1	1
mmu-miR-125b-5p	Map3k9	3UTR	1	1
mmu-miR-125b-5p	Grb10	3UTR	1	1
mmu-miR-125b-5p	Homez	3UTR	1	1
mmu-miR-125b-5p	Phactr3	3UTR	1	1
mmu-miR-125b-5p	Lclat1	3UTR	1	1
mmu-miR-125b-5p	Golga5	3UTR	1	1
mmu-miR-125b-5p	Trp53inp1	3UTR	1	1
mmu-miR-125b-5p	Reep3	3UTR	1	1
mmu-miR-125b-5p	Reep3	3UTR	1	1
mmu-miR-125b-5p	Scarb1	3UTR	1	1
mmu-miR-125b-5p	Slc4a10	3UTR	1	1
mmu-miR-125b-5p	Slc4a10	3UTR	1	1
mmu-miR-125b-5p	Tjap1	3UTR	1	1
mmu-miR-125b-5p	Tjap1	3UTR	1	1
mmu-miR-125b-5p	Tjap1	3UTR	1	1
mmu-miR-125b-5p	St6gal1	3UTR	1	1
mmu-miR-125b-5p	St6gal1	3UTR	1	1
mmu-miR-125b-5p	St6gal1	3UTR	1	1
mmu-miR-125b-5p	St6gal1	3UTR	1	1
mmu-miR-125b-5p	Cbll1	3UTR	1	1
mmu-miR-125b-5p	Cbll1	3UTR	1	1
mmu-miR-125b-5p	Cbll1	3UTR	1	1
mmu-miR-125b-5p	Cbll1	3UTR	1	1
mmu-miR-125b-5p	Fut1	3UTR	1	1
mmu-miR-125b-5p	Fut1	3UTR	1	1
mmu-miR-125b-5p	Cacnb1	3UTR	1	1
mmu-miR-125b-5p	Fmn1	3UTR	1	1
mmu-miR-125b-5p	Wnt11	3UTR	1	1
mmu-miR-125b-5p	Wnt11	3UTR	1	1
mmu-miR-125b-5p	C77080	3UTR	1	1
mmu-miR-125b-5p	C77080	3UTR	1	1
mmu-miR-125b-5p	C77080	3UTR	1	1
mmu-miR-125b-5p	C77080	3UTR	1	1
mmu-miR-125b-5p	Nfib	3UTR	1	1
mmu-miR-125b-5p	Nfib	3UTR	1	1
mmu-miR-125b-5p	Mtus1	3UTR	1	1
mmu-miR-125b-5p	Mtus1	3UTR	1	1
mmu-miR-125b-5p	Daam1	3UTR	1	1
mmu-miR-125b-5p	Fam131b	3UTR	1	1
mmu-miR-125b-5p	Dram2	3UTR	1	1
mmu-miR-125b-5p	Arid3a	3UTR	1	1
mmu-miR-125b-5p	Arid3a	3UTR	1	1
mmu-miR-125b-5p	Tbc1d1	3UTR	1	1
mmu-miR-125b-5p	St6galnac6	3UTR	1	1
mmu-miR-125b-5p	St6galnac6	3UTR	1	1
mmu-miR-125b-5p	St6galnac6	3UTR	1	1
mmu-miR-125b-5p	Asic1	3UTR	1	1
mmu-miR-125b-5p	Rora	3UTR	1	1
mmu-miR-125b-5p	Rora	3UTR	1	1
mmu-miR-125b-5p	Rorb	3UTR	1	1
mmu-miR-125b-5p	Map3k10	3UTR	1	1
mmu-miR-125b-5p	Acer2	3UTR	1	1
mmu-miR-125b-5p	Acer2	3UTR	1	1

mmu-miR-125b-5p	Suv39h1	3UTR	1	1
mmu-miR-125b-5p	Zscan29	3UTR	1	1
mmu-miR-125b-5p	Zscan29	3UTR	1	1
mmu-miR-125b-5p	Kcnip3	3UTR	1	1
mmu-miR-125b-5p	Kcnip3	3UTR	1	1
mmu-miR-125b-5p	Pbx1	3UTR	1	1
mmu-miR-125b-5p	Pbx1	3UTR	1	1
mmu-miR-125b-5p	Map2k7	3UTR	1	1
mmu-miR-125b-5p	Unc5c	3UTR	1	1
mmu-miR-125b-5p	Slc6a17	3UTR	1	1
mmu-miR-125b-5p	Zfp827	3UTR	1	1
mmu-miR-125b-5p	Zfp827	3UTR	1	1
mmu-miR-125b-5p	Tmem132e	3UTR	1	1
mmu-miR-125b-5p	Tmem132e	3UTR	1	1
mmu-miR-125b-5p	Car12	3UTR	1	1
mmu-miR-125b-5p	Trps1	3UTR	1	1
mmu-miR-125b-5p	Lurap1l	3UTR	1	1
mmu-miR-125b-5p	Triap1	3UTR	1	1
mmu-miR-125b-5p	Bap1	3UTR	1	1
mmu-miR-125b-5p	Khnyn	3UTR	1	1
mmu-miR-125b-5p	Lrfn2	3UTR	1	1
mmu-miR-125b-5p	Ankrd33b	3UTR	1	1
mmu-miR-125b-5p	Ankrd33b	3UTR	1	1
mmu-miR-125b-5p	Ankrd33b	3UTR	1	1
mmu-miR-125b-5p	Galnt14	3UTR	1	1
mmu-miR-125b-5p	Tmem25	3UTR	1	1
mmu-miR-125b-5p	Slc46a3	3UTR	1	1
mmu-miR-125b-5p	Slc46a3	3UTR	1	1
mmu-miR-125b-5p	Ddx42	3UTR	1	1
mmu-miR-125b-5p	Ppme1	3UTR	1	1
mmu-miR-125b-5p	Zfp518a	3UTR	1	1
mmu-miR-125b-5p	Prrc1	3UTR	1	1
mmu-miR-125b-5p	Ttc7	3UTR	1	1
mmu-miR-125b-5p	Pi4k2b	3UTR	1	1
mmu-miR-125b-5p	Tjap1	3UTR	1	1
mmu-miR-125b-5p	Gga2	3UTR	1	1
mmu-miR-125b-5p	Phactr3	3UTR	1	1
mmu-miR-125b-5p	Mtmr3	3UTR	1	1
mmu-miR-125b-5p	Retreg3	3UTR	1	1
mmu-miR-125b-5p	Retreg3	3UTR	1	1
mmu-miR-125b-5p	Ppp6r3	3UTR	1	1
mmu-miR-125b-5p	Mfsd13a	3UTR	1	1
mmu-miR-125b-5p	Klhl24	3UTR	1	1
mmu-miR-125b-5p	Smurf1	3UTR	1	1
mmu-miR-125b-5p	Smurf1	3UTR	1	1
mmu-miR-125b-5p	Ppp6r3	3UTR	1	1
mmu-miR-125b-5p	Fam131b	3UTR	1	1
mmu-miR-125b-5p	Tsen54	3UTR	1	1
mmu-miR-125b-5p	Kcnk10	3UTR	1	1
mmu-miR-125b-5p	Kcnk10	3UTR	1	1
mmu-miR-125b-5p	Lbh	3UTR	1	1
mmu-miR-125b-5p	Lbh	3UTR	1	1
mmu-miR-125b-5p	Zfp687	3UTR	1	1
mmu-miR-125b-5p	Avl9	3UTR	1	1

mmu-miR-125b-5p	Abtb1	3UTR	1	1
mmu-miR-125b-5p	Rad54l2	3UTR	1	1
mmu-miR-125b-5p	Rad54l2	3UTR	1	1
mmu-miR-125b-5p	Rad54l2	3UTR	1	1
mmu-miR-125b-5p	Ogfr	3UTR	1	1
mmu-miR-125b-5p	Cnnm1	3UTR	1	1
mmu-miR-125b-5p	Trps1	3UTR	1	1
mmu-miR-125b-5p	Trps1	3UTR	1	1
mmu-miR-125b-5p	D17H6S53E	3UTR	1	1
mmu-miR-125b-5p	Slc4a10	3UTR	1	1
mmu-miR-125b-5p	Pip4k2b	3UTR	1	1
mmu-miR-125b-5p	Baz2a	3UTR	1	1
mmu-miR-125b-5p	Alpk3	3UTR	1	1
mmu-miR-125b-5p	Sertad3	3UTR	1	1
mmu-miR-125b-5p	Cd34	3UTR	1	1
mmu-miR-125b-5p	Tmem123	3UTR	1	1
mmu-miR-125b-5p	Osbpl9	3UTR	1	1
mmu-miR-125b-5p	AU040320	3UTR	1	1
mmu-miR-125b-5p	Ncln	3UTR	1	1
mmu-miR-125b-5p	Cbll1	3UTR	1	1
mmu-miR-125b-5p	Rnf44	3UTR	1	1
mmu-miR-125b-5p	Fam83h	3UTR	1	1
mmu-miR-125b-5p	Nrm	3UTR	1	1
mmu-miR-125b-5p	Bmf	3UTR	1	1
mmu-miR-125b-5p	Bmf	3UTR	1	1
mmu-miR-125b-5p	Edem1	3UTR	1	1
mmu-miR-125b-5p	Rassf3	3UTR	1	1
mmu-miR-125b-5p	Rassf3	3UTR	1	1
mmu-miR-125b-5p	Atxn7	3UTR	1	1
mmu-miR-125b-5p	Acer2	3UTR	1	1
mmu-miR-125b-5p	Acer2	3UTR	1	1
mmu-miR-125b-5p	Cep85	3UTR	1	1
mmu-miR-125b-5p	Galnt7	3UTR	1	1
mmu-miR-125b-5p	Ggt7	3UTR	1	1
mmu-miR-125b-5p	Ggt7	3UTR	1	1
mmu-miR-125b-5p	Cacnb1	3UTR	1	1
mmu-miR-125b-5p	Mlf2	3UTR	1	1
mmu-miR-125b-5p	Mlf2	3UTR	1	1
mmu-miR-125b-5p	Mark1	3UTR	1	1
mmu-miR-125b-5p	Rap1a	3UTR	1	1
mmu-miR-125b-5p	Eva1a	3UTR	1	1
mmu-miR-125b-5p	Lin28a	3UTR	1	1
mmu-miR-125b-5p	Lin28a	3UTR	1	1
mmu-miR-125b-5p	St6gal1	3UTR	1	1
mmu-miR-125b-5p	St6gal1	3UTR	1	1
mmu-miR-125b-5p	Dennd6a	3UTR	1	1
mmu-miR-125b-5p	Rhot2	3UTR	1	1
mmu-miR-125b-5p	Rorb	3UTR	1	1
mmu-miR-125b-5p	Niban2	3UTR	1	1
mmu-miR-125b-5p	Kctd15	3UTR	1	1
mmu-miR-125b-5p	Slc8a2	3UTR	1	1
mmu-miR-125b-5p	Sirt7	3UTR	1	1
mmu-miR-125b-5p	Mapre2	3UTR	1	1
mmu-miR-125b-5p	Ppp1r16b	3UTR	1	1

mmu-miR-125b-5p	Dcaf10	3UTR	1	1
mmu-miR-125b-5p	Dcaf10	3UTR	1	1
mmu-miR-125b-5p	Dusp7	3UTR	1	1
mmu-miR-125b-5p	Dis3l2	3UTR	1	1
mmu-miR-125b-5p	Fam20a	3UTR	1	1
mmu-miR-125b-5p	Glb1l2	3UTR	1	1
mmu-miR-125b-5p	Slc6a17	3UTR	1	1
mmu-miR-125b-5p	Stxbp5l	3UTR	1	1
mmu-miR-125b-5p	Stxbp5l	3UTR	1	1
mmu-miR-125b-5p	Dtx4	3UTR	1	1
mmu-miR-125b-5p	Tbc1d16	3UTR	1	1
mmu-miR-125b-5p	Tbc1d16	3UTR	1	1
mmu-miR-125b-5p	Daam1	3UTR	1	1
mmu-miR-125b-5p	Zdhhc9	3UTR	1	1
mmu-miR-125b-5p	Mfsd9	3UTR	1	1
mmu-miR-125b-5p	Fam126b	3UTR	1	1
mmu-miR-125b-5p	Fam126b	3UTR	1	1
mmu-miR-125b-5p	Nipal4	3UTR	1	1
mmu-miR-125b-5p	Tdg	3UTR	1	1
mmu-miR-125b-5p	Zfp523	3UTR	1	1
mmu-miR-125b-5p	Dpp9	3UTR	1	1
mmu-miR-125b-5p	Lrp4	3UTR	1	1
mmu-miR-125b-5p	Lrp4	3UTR	1	1
mmu-miR-125b-5p	Samd10	3UTR	1	1
mmu-miR-125b-5p	E130309D02Rik	3UTR	1	1
mmu-miR-125b-5p	Tbx4	3UTR	1	1
mmu-miR-125b-5p	Ccnj	3UTR	1	1
mmu-miR-125b-5p	Galnt5	3UTR	1	1
mmu-miR-125b-5p	Cops7b	3UTR	1	1
mmu-miR-125b-5p	Cops7b	3UTR	1	1
mmu-miR-125b-5p	Tspan12	3UTR	1	1
mmu-miR-125b-5p	Myo9a	3UTR	1	1
mmu-miR-125b-5p	Fndc3b	3UTR	1	1
mmu-miR-125b-5p	Osbpl9	3UTR	1	1
mmu-miR-125b-5p	Gcnt1	3UTR	1	1
mmu-miR-125b-5p	Gcnt1	3UTR	1	1
mmu-miR-125b-5p	Vcpip1	3UTR	1	1
mmu-miR-125b-5p	Rab6b	3UTR	1	1
mmu-miR-125b-5p	Rab6b	3UTR	1	1
mmu-miR-125b-5p	Fam78b	3UTR	1	1
mmu-miR-125b-5p	Fam78b	3UTR	1	1
mmu-miR-125b-5p	Prtg	3UTR	1	1
mmu-miR-125b-5p	Slitrk6	3UTR	1	1
mmu-miR-125b-5p	Hif1an	3UTR	1	1
mmu-miR-125b-5p	Usp37	3UTR	1	1
mmu-miR-125b-5p	Tmem198	3UTR	1	1
mmu-miR-125b-5p	Msrb3	3UTR	1	1
mmu-miR-125b-5p	Msrb3	3UTR	1	1
mmu-miR-125b-5p	Tmtc2	3UTR	1	1
mmu-miR-125b-5p	Map3k9	3UTR	1	1
mmu-miR-125b-5p	Prkaa2	3UTR	1	1
mmu-miR-125b-5p	Zfp827	3UTR	1	1
mmu-miR-125b-5p	Zfp827	3UTR	1	1
mmu-miR-125b-5p	Car12	3UTR	1	1

mmu-miR-125b-5p	Gpr153	3UTR	1	1
mmu-miR-125b-5p	Reep3	3UTR	1	1
mmu-miR-125b-5p	Slc38a9	3UTR	1	1
mmu-miR-125b-5p	Hs3st3a1	3UTR	1	1
mmu-miR-125b-5p	Hic2	3UTR	1	1
mmu-miR-125b-5p	Slc25a15	3UTR	1	1
mmu-miR-125b-5p	Zfyve1	3UTR	1	1
mmu-miR-125b-5p	Zfyve1	3UTR	1	1
mmu-miR-125b-5p	Zfyve1	3UTR	1	1
mmu-miR-125b-5p	Homez	3UTR	1	1
mmu-miR-125b-5p	Nbeal2	3UTR	1	1
mmu-miR-125b-5p	Pbx1	3UTR	1	1
mmu-miR-125b-5p	Wipf2	3UTR	1	1
mmu-miR-125b-5p	Wipf2	3UTR	1	1
mmu-miR-125b-5p	Usp2	3UTR	1	1
mmu-miR-125b-5p	Usp2	3UTR	1	1
mmu-miR-125b-5p	Dagla	3UTR	1	1
mmu-miR-125b-5p	Zfp395	3UTR	1	1
mmu-miR-125b-5p	Rusc2	3UTR	1	1
mmu-miR-125b-5p	Jade2	3UTR	1	1
mmu-miR-125b-5p	Jade2	3UTR	1	1
mmu-miR-125b-5p	Ece1	3UTR	1	1
mmu-miR-125b-5p	Ece1	3UTR	1	1
mmu-miR-125b-5p	Fgfr2	3UTR	1	1
mmu-miR-125b-5p	Ninl	3UTR	1	1
mmu-miR-125b-5p	Ppp4r3a	3UTR	1	1
mmu-miR-125b-5p	Cacna1b	3UTR	1	1
mmu-miR-125b-5p	Cacna1b	3UTR	1	1
mmu-miR-125b-5p	Osbpl9	3UTR	1	1
mmu-miR-125b-5p	Glb1l2	3UTR	1	1
mmu-miR-125b-5p	Usp2	3UTR	1	1
mmu-miR-125b-5p	Usp2	3UTR	1	1
mmu-miR-125b-5p	Myo9a	3UTR	1	1
mmu-miR-125b-5p	Zbtb7a	3UTR	1	1
mmu-miR-125b-5p	Mtmr3	3UTR	1	1
mmu-miR-125b-5p	Ubn1	3UTR	1	1
mmu-miR-125b-5p	Ube2l3	3UTR	1	1
mmu-miR-125b-5p	Hnf4g	3UTR	1	1
mmu-miR-125b-5p	Tbc1d1	3UTR	1	1
mmu-miR-125b-5p	Baz2a	3UTR	1	1
mmu-miR-125b-5p	Zfp523	3UTR	1	1
mmu-miR-125b-5p	Eva1a	3UTR	1	1
