# Supplementary material for: LncRNA AK089514/miR-125b-5p/TRAF6 axis mediates macrophage polarization in allergic asthma
Source: BMC Pulm Med. 2023 Jan 30;23:45. doi: 10.1186/s12890-023-02339-1 (PMC9887860; doi:10.1186/s12890-023-02339-1)
Supplement: Supplementary file 4 — Additional file 4: Table S3. lncRNA target miR-125b-5p. [file 12890_2023_2339_MOESM4_ESM.pdf]

Table S3. lncRNA target miR-125b-5p

ProbeName	Regulation	Classify	Mirbase ID	Site Type
CUST_81209_PI428753551	up	lncRNA	mmu-miR-125b-5p	7mer-1a
CUST_80261_PI428753551	up	lncRNA	mmu-miR-125b-5p	7mer-1a
CUST_28779_PI428753551	up	lncRNA	mmu-miR-125b-5p	8mer-1a
CUST_8595_PI428753551	up	lncRNA_mRNA	mmu-miR-125b-5p	7mer-1a
CUST_9717_PI428753551	up	lncRNA	mmu-miR-125b-5p	7mer-1a
CUST_9718_PI428753551	up	lncRNA	mmu-miR-125b-5p	7mer-1a
CUST_81924_PI428753551	up	lncRNA_mRNA	mmu-miR-125b-5p	8mer-1a
CUST_80259_PI428753551	up	lncRNA	mmu-miR-125b-5p	7mer-1a
CUST_4489_PI428753551	up	lncRNA	mmu-miR-125b-5p	7mer-1a
CUST_71807_PI428753551	up	lncRNA	mmu-miR-125b-5p	7mer-1a
CUST_41314_PI428753551	up	lncRNA	mmu-miR-125b-5p	7mer-1a
CUST_38517_PI428753551	up	lncRNA_mRNA	mmu-miR-125b-5p	7mer-1a
CUST_41313_PI428753551	up	lncRNA	mmu-miR-125b-5p	7mer-1a
CUST_4490_PI428753551	up	lncRNA	mmu-miR-125b-5p	7mer-1a
CUST_59533_PI428753551	up	lncRNA	mmu-miR-125b-5p	7mer-m8
CUST_3270_PI428753551	up	lncRNA_mRNA	mmu-miR-125b-5p	7mer-1a
CUST_3268_PI428753551	up	lncRNA_mRNA	mmu-miR-125b-5p	7mer-1a
CUST_75444_PI428753551	up	lncRNA	mmu-miR-125b-5p	8mer-1a
CUST_60471_PI428753551	up	lncRNA	mmu-miR-125b-5p	8mer-1a
CUST_72638_PI428753551	up	lncRNA	mmu-miR-125b-5p	7mer-1a
CUST_72639_PI428753551	up	lncRNA	mmu-miR-125b-5p	7mer-1a
CUST_58966_PI428753551	up	lncRNA	mmu-miR-125b-5p	7mer-1a
CUST_60443_PI428753551	up	lncRNA	mmu-miR-125b-5p	7mer-m8
CUST_81923_PI428753551	up	lncRNA_mRNA	mmu-miR-125b-5p	8mer-1a
CUST_3980_PI428753551	up	lncRNA	mmu-miR-125b-5p	7mer-1a
CUST_57957_PI428753551	up	lncRNA	mmu-miR-125b-5p	7mer-m8
CUST_80651_PI428753551	up	lncRNA	mmu-miR-125b-5p	7mer-m8
CUST_17455_PI428753551	up	lncRNA	mmu-miR-125b-5p	8mer-1a
CUST_3269_PI428753551	up	lncRNA_mRNA	mmu-miR-125b-5p	7mer-1a
CUST_38356_PI428753551	up	lncRNA_mRNA	mmu-miR-125b-5p	7mer-m8
CUST_17457_PI428753551	up	lncRNA	mmu-miR-125b-5p	8mer-1a
CUST_33846_PI428753551	up	lncRNA	mmu-miR-125b-5p	7mer-m8
CUST_80798_PI428753551	up	lncRNA	mmu-miR-125b-5p	7mer-m8
CUST_15855_PI428753551	up	lncRNA	mmu-miR-125b-5p	7mer-1a
CUST_38357_PI428753551	up	lncRNA_mRNA	mmu-miR-125b-5p	7mer-m8
CUST_46859_PI428753551	up	lncRNA	mmu-miR-125b-5p	7mer-1a
CUST_25848_PI428753551	up	lncRNA	mmu-miR-125b-5p	7mer-1a
CUST_59944_PI428753551	up	lncRNA	mmu-miR-125b-5p	7mer-1a
CUST_35223_PI428753551	up	lncRNA	mmu-miR-125b-5p	7mer-1a
CUST_21755_PI428753551	up	lncRNA	mmu-miR-125b-5p	7mer-m8
CUST_51399_PI428753551	up	lncRNA	mmu-miR-125b-5p	8mer-1a
CUST_80260_PI428753551	up	lncRNA	mmu-miR-125b-5p	7mer-1a
CUST_65021_PI428753551	up	lncRNA_mRNA	mmu-miR-125b-5p	7mer-m8
CUST_10203_PI428753551	up	lncRNA_mRNA	mmu-miR-125b-5p	7mer-m8
CUST_38516_PI428753551	up	lncRNA_mRNA	mmu-miR-125b-5p	7mer-1a
CUST_26237_PI428753551	up	lncRNA	mmu-miR-125b-5p	7mer-m8
CUST_81913_PI428753551	up	lncRNA_mRNA	mmu-miR-125b-5p	7mer-1a
CUST_6840_PI428753551	up	lncRNA_mRNA	mmu-miR-125b-5p	7mer-1a
CUST_57707_PI428753551	up	lncRNA_mRNA	mmu-miR-125b-5p	8mer-1a
CUST_75474_PI428753551	up	lncRNA	mmu-miR-125b-5p	7mer-m8

CUST_67019_PI428753551	up	lncRNA	mmu-miR-125b-5p	7mer-m8
CUST_6842_PI428753551	up	lncRNA_mRNA	mmu-miR-125b-5p	7mer-1a
CUST_81915_PI428753551	up	lncRNA_mRNA	mmu-miR-125b-5p	7mer-1a
CUST_2826_PI428753551	up	lncRNA	mmu-miR-125b-5p	7mer-1a
CUST_51400_PI428753551	up	lncRNA	mmu-miR-125b-5p	8mer-1a
CUST_48462_PI428753551	up	lncRNA	mmu-miR-125b-5p	8mer-1a
CUST_1029_PI428753551	up	lncRNA	mmu-miR-125b-5p	7mer-m8
CUST_8356_PI428753551	up	lncRNA_mRNA	mmu-miR-125b-5p	7mer-1a
CUST_57706_PI428753551	up	lncRNA_mRNA	mmu-miR-125b-5p	8mer-1a
CUST_8357_PI428753551	up	lncRNA_mRNA	mmu-miR-125b-5p	7mer-1a
CUST_67018_PI428753551	up	lncRNA	mmu-miR-125b-5p	7mer-m8
CUST_67020_PI428753551	up	lncRNA	mmu-miR-125b-5p	7mer-m8
CUST_76153_PI428753551	up	lncRNA	mmu-miR-125b-5p	8mer-1a
CUST_22091_PI428753551	up	lncRNA_mRNA	mmu-miR-125b-5p	7mer-m8
CUST_78942_PI428753551	up	lncRNA_mRNA	mmu-miR-125b-5p	7mer-m8
CUST_3907_PI428753551	up	lncRNA_mRNA	mmu-miR-125b-5p	7mer-m8
CUST_79026_PI428753551	up	lncRNA	mmu-miR-125b-5p	7mer-1a
CUST_31749_PI428753551	up	lncRNA	mmu-miR-125b-5p	8mer-1a
CUST_6841_PI428753551	up	lncRNA_mRNA	mmu-miR-125b-5p	7mer-1a
CUST_34884_PI428753551	up	lncRNA	mmu-miR-125b-5p	7mer-m8
CUST_79030_PI428753551	up	lncRNA_mRNA	mmu-miR-125b-5p	7mer-m8
CUST_52711_PI428753551	up	lncRNA_mRNA	mmu-miR-125b-5p	7mer-1a
CUST_27709_PI428753551	up	lncRNA	mmu-miR-125b-5p	7mer-m8
CUST_33917_PI428753551	up	lncRNA	mmu-miR-125b-5p	7mer-1a
CUST_57708_PI428753551	up	lncRNA_mRNA	mmu-miR-125b-5p	8mer-1a
CUST_79072_PI428753551	up	lncRNA_mRNA	mmu-miR-125b-5p	7mer-1a
CUST_31615_PI428753551	up	lncRNA	mmu-miR-125b-5p	7mer-1a
CUST_38759_PI428753551	up	lncRNA	mmu-miR-125b-5p	7mer-m8
CUST_78943_PI428753551	up	lncRNA_mRNA	mmu-miR-125b-5p	7mer-m8
CUST_79070_PI428753551	up	lncRNA_mRNA	mmu-miR-125b-5p	7mer-1a
CUST_78978_PI428753551	up	lncRNA_mRNA	mmu-miR-125b-5p	7mer-1a
CUST_17387_PI428753551	up	lncRNA_mRNA	mmu-miR-125b-5p	8mer-1a
